# Synthesis and Anticancer Activities of Pyrazole–Thiadiazole-Based EGFR Inhibitors

**DOI:** 10.1021/acsomega.3c04635

**Published:** 2023-08-17

**Authors:** Berkant Kurban, Begüm Nurpelin Sağlık, Derya Osmaniye, Serkan Levent, Yusuf Özkay, Zafer Asım Kaplancıklı

**Affiliations:** † Department of Pharmaceutical Chemistry, Faculty of Pharmacy, Afyonkarahisar Health Sciences University, Afyonkarahisar 03030, Turkey; ‡ Department of Pharmaceutical Chemistry, Faculty of Pharmacy, Anadolu University, Eskişehir 26470, Turkey; § Central Research Laboratory (MERLAB), Faculty of Pharmacy, Anadolu University, Eskişehir 26470, Turkey

## Abstract

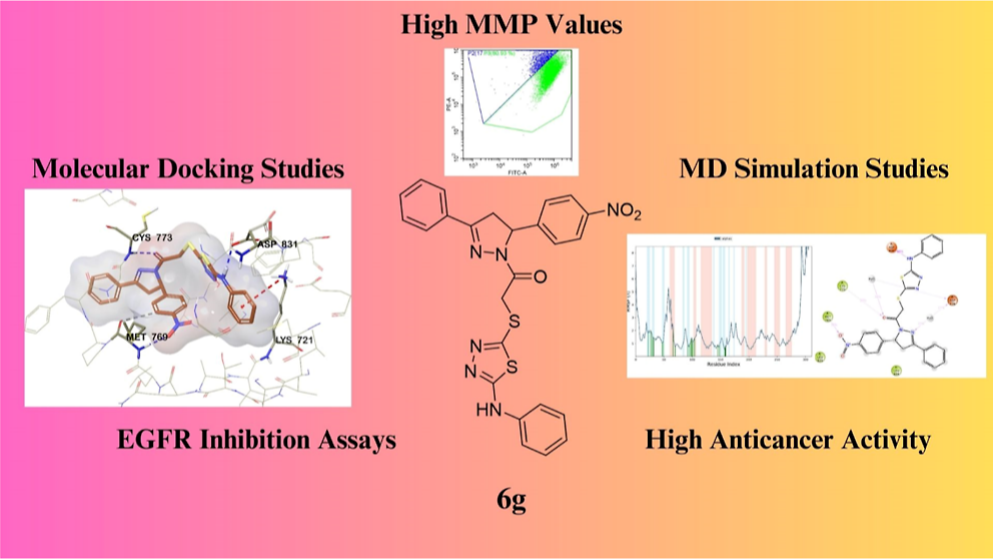

Lung cancer is one of the most common cancer types of cancer with the highest mortality rates. However, while epidermal growth factor receptor (EGFR) is an important parameter for lung cancer, EGFR inhibitors also show great promise in the treatment of the disease. Therefore, a series of new EGFR inhibitor candidates containing thiadiazole and pyrazole rings have been developed. The activities of the synthesized compounds were elucidated by in vitro MTT, (which is chemically 3-(4,5-dimethylthiazol-2-yl)-2,5-diphenyltetrazolium bromide), cytotoxicity assay, analysis of mitochondrial membrane potential (MMP) by flow cytometry, and EGFR inhibition experiments. Molecular docking and molecular dynamics simulations were performed as in silico studies. Compounds **6d**, **6g**, and **6j** showed inhibitor activity against the A549 cell line with IC_50_ = 5.176 ± 0.164; 1.537 ± 0.097; and 8.493 ± 0.667 μM values, respectively. As a result of MMP by flow cytometry, compound **6g** showed 80.93% mitochondrial membrane potential. According to the results of the obtained EGFR inhibitory assay, compound **6g** shows inhibitory activity on the EGFR enzyme with a value of IC_50_ = 0.024 ± 0.002 μM.

## 1 Introduction

Cancer is one of the most serious and deadly health problems. Lung cancer, on the other hand, is quite deadly compared to other cancer subtypes.^1,2^ Kinases are enzymes responsible for phosphate transfer. Cytoplasmic tyrosine kinases, a subgroup of kinases, are critical for extracellular signals. These extracellular signals have been shown to occur in a variety of oncogenic conditions. Naturally, the development of tyrosine kinase enzyme inhibitors is very important in cancer treatment.^3,4^

Epidermal growth factor receptors (EGFRs), one of the tyrosine kinase receptors, are responsible for cell growth and proliferation. However, abnormal conditions in EGFR functions cause cancer. Therefore, EGFR inhibitors are very important for new drug discovery in the treatment of certain types of cancer, such as lung cancer and triple-negative breast cancer.^5,6^

Heterocyclic compounds are known to have anticancer activity.^7−9^ An important example of the heterocyclic compounds is Erlotinib. Erlotinib is an FDA-approved inhibitor of EGFR.^10^ Pyrazolines, which contain a double bond and two adjacent nitrogen atoms in the ring, are five-membered heterocyclic rings and show anticancer activity.^11,12^ Furthermore, the EGFR inhibition values shown by the compounds containing the 1,3,5-trisubstituted pyrazoline structure have been a guide in the design process of the compounds.^13,14^ In previous studies, it was also observed that compounds containing a pyrazole ring had very good inhibition values on EGFR.^15,16^ Just like the pyrazole ring, the thiadiazole ring has anticancer activity. Studies have shown that compounds containing a thiadiazole ring are pretentious about anticancer activity.^17−19^ Thiadiazoles also showed anticancer activity by providing EGFR inhibition.^20,21^

**Figure 1:**
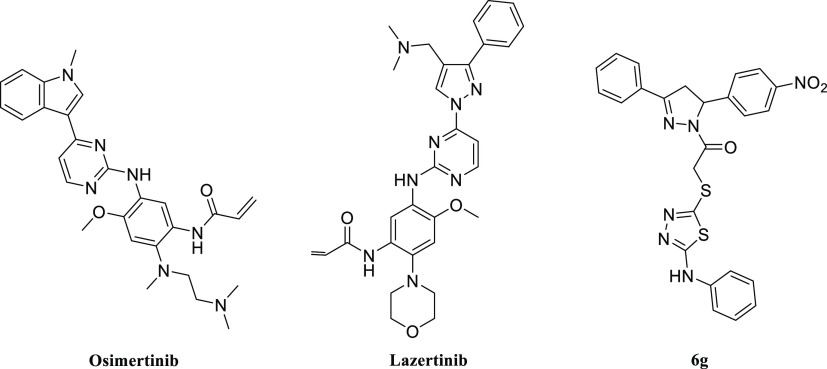
Third-generation EGFR inhibitors (Osimertinib–Lazertinib) and compound **6g**.

In the studies on several compounds containing thiazole, a thiadiazole-like heterocyclic ring, and a pyrazole ring, it has been determined that the compounds are anticancer drug candidates with a very high EGFR inhibition potential.^22−26^ Lazertinib, a third-generation EGFR inhibitor, has had promising results in patients who have not yet started treatment or in patients with Osimertinib resistance. Lazertinib is unique and different from other third-generation EGFR inhibitors in that it contains a pyrazole ring, heterocyclic rings, hydrophobic phenyl, and hydrophilic amine structures. As seen in Figure 1, the chemical structures of the compounds are like Lasertinib.^27,28^

In light of all this information, the hybrid compounds including pyrazole–thiadiazol rings were designed as EGFR inhibitors.

## 2 Experimental Section

### 2.1 Chemistry

All the chemicals were obtained from industrial vendors and used without additional purification. Melting points (mp) were determined on the Mettler Toledo-MP90 Melting Point System. ^1^H-NMR Bruker DPX 300 FT-NMR spectrometer and ^13^C-NMR, Bruker DPX 75 MHz spectrometer (Bruker Bioscience, Billerica, MA, USA) were used for NMR analysis. Mass spectra were recorded on an LCMS-IT-TOF (Shimadzu, Kyoto, Japan) using ESI.

#### 2.1.1 General Procedure for the Synthesis of the Compounds

##### 2.1.1.1 Synthesis of 3-(4-Nitrophenyl)-1-phenylprop-2-en-1-one (**1**)

KOH solution (40%) was prepared in MeOH (>99.5%). Acetophenone (1.201 g, 0.010 mol) and the prepared KOH solution were stirred at room temperature for 30 min. Then, 4-nitrobenzaldehyde (1.510 g, 0.010 mol) was added to the reaction and mixed. After the reaction was complete, the precipitated product was filtered, washed with MeOH, and dried.^29^

##### 2.1.1.2 Synthesis of 5-(4-Nitrophenyl)-3-phenyl-4,5-dihydro-1*H*-pyrazole (**2**)

3-(4-Nitrophenyl)-1-phenylprop-2-en-1-one (1.265 g, 0.005 mol) was dissolved in ethanol (30 mL) and refluxed with hydrazine hydrate (0.501 g, 0.010 mol) for 8 h. After the reaction was complete, the reaction mixture was cooled and refrigerated overnight. The resulting solid was filtered, and the crude pyrazoline was dried and recrystallized from ethanol.^30^

##### 2.1.1.3 Synthesis of 2-Chloro-1-(5-(4-nitrophenyl)-3-phenyl-4,5-dihydro-1*H*-pyrazole-1-yl)ethan-1-one (**3**)

First, 5-(4-nitrophenyl)-3-phenyl-4,5-dihydro-1*H*-pyrazole (0.534 g, 0.002 mol) was dissolved in 100 mL of tetrahydrofuran (THF), and triethylamine (TEA) was added to the solution. Then, chloroacetyl chloride dissolved in 10 mL of THF was added dropwise to the solution placed in the ice bath because of the obtained 2-chloro-1-(5-(4-nitrophenyl)-3-phenyl-4,5-dihydro-1*H*-pyrazole-1-yl)ethan-1-one (3).

##### 2.1.1.4 Synthesis of Compounds **4a–4j**

An excess of hydrazine hydrate was added to the mixture of alkyl/aryl isothiocyanates (0.010 mol) in ethanol (EtOH; 40 mL) and stirred in an ice bath for 2 h. After the reaction was finished, the precipitated product was filtered and washed with cold EtOH. The product was then dried and recrystallized from EtOH.^31^

##### 2.1.1.5 Synthesis of Thiadiazoles (**5a–5j**)

First, NaOH (0.2 g, 0.005 mol) was dissolved in 40 mL of ethanol, and compounds **4a–4j** (0.005 mol) were added. Then, carbon disulfide (0.36 mL, 0.006 mol) was added to the mixture and refluxed for 4 h. After the reaction was complete, the mixture was poured into ice water and acidified to pH 4–5 with 20% HCl. The precipitated product was filtered and washed with water. The product was then dried and recrystallized from EtOH.^31^

##### 2.1.1.6 Synthesis of the Target Compounds (**6a–6j**)

First, thiadiazole derivatives (**5a–5j**) (0.001 mol) and compound **3** (0.343 g, 0.001 mol) were dissolved in 20 mL of acetone. 0.001 mol of K_2_CO_3_ was then added, and the mixture was refluxed overnight. After the reaction was complete, the mixture was cooled, and the precipitated product was filtered and purified by recrystallization with ethanol.^32^

2-((5-(Ethylamino)-1,3,4-thiadiazol-2-yl)thio)-1-(5-(4-nitrophenyl)-3-phenyl-4,5-dihydro-1*H*-pyrazol-1-yl)ethan-1-one (**6a**): yield: 81%, mp: 161.7–162.3 °C. ^1^H-NMR (300 MHz, DMSO-*d*_6_): δ = 1.17 (3H, t, *J* = 7.19 Hz, −CH_3_), 3.26 (2H, m, −CH_2_), 3.24 (2H, m, −CH_2_), 3.32 (1H, m, pyrazole), 4.01 (1H, dd, *J*_1_ = 12.01 Hz, *J*_2_ = 18.34 Hz, pyrazole), 4.46 (2H, m, −CH_2_), 5.79 (1H, dd, *J*_1_ = 5.07 Hz, *J*_2_ = 11.95 Hz, pyrazole), 7.52 (3H, m, monosubstituted benzene), 7.60 (2H, d, *J* = 8.83 Hz, disubstituted benzene), 7.83 (2H, d, *J* = 2.55 Hz, monosubstituted benzene) 7.85 (1H, s, seconder amine), 8.25 (2H, d, *J* = 8.77 Hz , disubstituted benzene). ^13^C-NMR (75 MHz, DMSO-*d*_6_): δ = 14.60 (ethyl-C), 37.29 (−CH_2_−), 39.83 (ethyl-C), 42.29 (pyrazole-C), 59.87 (pyrazole-C), 124.37 (phenyl-C), 127.40 (phenyl-C), 127.56 (phenyl-C), 129.27 (phenyl-C), 130.94 (phenyl-C), 131.19 (phenyl-C), 147.18 (phenyl-C), 148.96 (phenyl-C), 149.47 (pyrazole-C), 155.71 (thiadiazole-C), 165.34 (thiadiazole-C), 170.32 (C=O). HRMS (*m*/*z*): [M + H]^+^ calcd for C_21_H_20_N_6_O_3_S_2_, 469.1111; found, 469.1106.

1-(5-(4-Nitrophenyl)-3-phenyl-4,5-dihydro-1*H*-pyrazol-1-yl)-2-((5-(propylamino)-1,3,4-thiadiazol-2-yl)thio)ethan-1-one (**6b**): yield: 72%, mp: 120.6–122.8 °C. ^1^H-NMR (300 MHz, DMSO-*d*_6_): δ = 0.87 (1H, m, −CH_3_), 1.52 (4H, m, −CH_2_CH_2_), 3.26 (1H, m, pyrazole), 3.94 (1H, dd, *J*_1_ = 14.01 Hz, *J*_2_ = 19.19 Hz, pyrazole), 4.40 (2H, m, −CH_2_), 5.73 (1H, dd, *J*_1_ = 5.04 Hz, *J*_2_ = 11.93 Hz, pyrazole), 7.46 (3H, m, monosubstituted benzene), 7.54 (2H, d, *J* = 8.80 Hz, disubstituted benzene), 7.78 (2H, d, *J* = 7.87 Hz, monosubstituted benzene), 7.82 (1H, s, seconder amine), 8.19 (2H, d, *J* = 8.78 Hz, disubstituted benzene). ^13^C-NMR (75 MHz, DMSO-*d*_6_): δ = 11.80 (propyl-C), 22.18 (propyl-C), 37.26 (−CH_2_), 42.29 (pyrazole-C), 46.82 (propyl-C), 59.87 (pyrazole-C), 124.36 (phenyl-C), 127.40 (phenyl-C), 127.56 (phenyl-C), 129.27 (phenyl-C), 130.94 (phenyl-C), 131.19 (phenyl-C), 147.18 (phenyl-C), 149.47 (phenyl-C), 155.70 (pyrazole-C), 165.35 (thiadiazole), 170.07 (thiadiazole-C), 170.50 (C=O). HRMS (*m*/*z*): [M + H]^+^ calcd for C_22_H_22_N_6_O_3_S_2_, 483.1268; found, 483.1265.

2-((5-(Isopropylamino)-1,3,4-thiadiazol-2-yl)thio)-1-(5-(4-nitrophenyl)-3-phenyl-4,5-dihydro-1*H*-pyrazol-1-yl)ethan-1-one (**6c**): yield: 82%, mp: 105.2–107.4 °C. ^1^H-NMR (300 MHz, DMSO-*d*_6_): δ = 1.13 (6H, dd, *J*_1_ = 1.77 Hz, *J*_2_ = 6.42 Hz, −CH_3_), 3.24 (1H, dd, *J*_1_ = 5.18 Hz, *J*_2_ = 18.48 Hz, pyrazole), 3.72 (1H, m, −CH), 3.95 (1H, dd, *J*_1_ = 12.04 Hz, *J*_2_ = 18.43 Hz, pyrazole), 4.41 (2H, m, −CH_2_), 5.73 (1H, dd, *J*_1_ = 5.04 Hz, *J*_2_ = 11.95 Hz, pyrazole), 7.46 (3H, m, monosubstituted benzene), 7.54 (2H, d, *J* = 8.83 Hz, disubstituted benzene), 7.72 (1H, d, *J* = 7.11 Hz, seconder amine), 7.81 (2H, m, monosubstituted benzene), 8.19 (2H, d, *J* = 8.80 Hz, disubstituted benzene). ^13^C-NMR (75 MHz, DMSO-*d*_6_): δ = 22.51 (isopropyl-C), 37.20 (−CH_2_−), 42.28 (pyrazole-C), 47.02 (isopropyl-C), 59.88 (pyrazole-C), 124.36 (phenyl-C), 127.40 (phenyl-C), 127.57 (phenyl-C), 129.28 (phenyl-C), 130.94 (phenyl-C), 131.20 (phenyl-C), 147.18 (phenyl-C), 148.85 (phenyl-C), 149.48 (phenyl-C), 155.70 (pyrazole-C), 165.34 (thiadiazole-C), 169.45 (C=O). HRMS (*m*/*z*): [M + H]^+^ calcd for C_22_H_22_N_6_O_3_S_2_, 483.1268; found, 483.1250.

2-((5-(Allylamino)-1,3,4-thiadiazol-2-yl)thio)-1-(5-(4-nitrophenyl)-3-phenyl-4,5-dihydro-1*H*-pyrazol-1-yl)ethan-1-one (**6d**): yield: 82%, mp: 118.5–120.6 °C. ^1^H-NMR (300 MHz, DMSO-*d*_6_): δ = 1.16 (1H, m, −CH), 1.83 (2H, m, −CH_2_), 3.02 (2H, m, −CH_2_), 3.23 (1H, dd, *J*_1_ = 5.12 Hz, *J*_2_ = 18.39 Hz, pyrazole), 3.72 (1H, m, −CH), 3.91 (1H, dd, *J*_1_ = 7.25 Hz, *J*_2_ = 19.26 Hz, pyrazole), 4.40 (2H, m, −CH_2_), 5.73 (1H, dd, *J*_1_ = 5.02 Hz, *J*_2_ = 11.92 Hz, pyrazole), 7.45 (3H, m, monosubstituted benzene), 7.54 (2H, d, *J* = 8.82 Hz, disubstituted benzene), 7.78 (2H, m, monosubstituted benzene), 7.85 (1H, s, seconder amine), 8.18 (2H, d, *J* = 8.79 Hz, disubstituted benzene). ^13^C-NMR (75 MHz, DMSO-*d*_6_): δ = 20.48 (−CH_2_−), 27.93 (pyrazole-C), 52.66 (allyl-C), 59.84 (pyrazole-C), 124.35 (allyl-C), 127.39 (phenyl-C), 127.55 (phenyl-C), 128.99 (phenyl-C), 129.27 (phenyl-C), 130.40 (allyl-C), 130.94 (phenyl-C), 131.19 (phenyl-C), 147.18 (phenyl-C), 148.83 (phenyl-C), 149.47 (phenyl-C), 155.69 (pyrazole-C), 165.35 (thiadiazole-C), 170.63 (C=O). HRMS (*m*/*z*): [M + H]^+^ calcd for C_22_H_20_N_6_O_3_S_2_, 481.1111; found, 481.1113.

2-((5-(Isopropylamino)-1,3,4-thiadiazol-2-yl)thio)-1-(5-(4-nitrophenyl)-3-phenyl-4,5-dihydro-1*H*-pyrazol-1-yl)ethan-1-one (**6e**): yield: 73%, mp: 123.7–126.4 °C. ^1^H-NMR (300 MHz, DMSO-*d*_6_): δ = 0.86 (1H, m, −CH_3_), 1.16 (2H, t, *J* = 7.28 Hz, −CH_2_), 1.31 (2H, t, *J* = 7.73 Hz, −CH_2_), 1.49 (2H, t, *J* = 7.47 Hz, −CH_2_), 3.17 (1H, dd, *J*_1_ = 1.44 Hz, *J*_2_ = 6.90 Hz, pyrazole), 3.94 (1H, dd, *J*_1_ = 14.95 Hz, *J*_2_ = 21.22 Hz, pyrazole), 4.40 (2H, m, −CH_2_), 5.73 (1H, dd, *J*_1_ = 5.09 Hz, *J*_2_ = 11.93 Hz, pyrazole), 7.46 (3H, m, monosubstituted benzene), 7.54 (2H, d, *J* = 8.86 Hz, disubstituted benzene), 7.77 (1H, d, *J* = 2.76 Hz, seconder amine), 7.83 (2H, m, monosubstituted benzene), 8.19 (2H, d, *J* = 8.83 Hz, disubstituted benzene). ^13^C-NMR (75 MHz, DMSO-*d*_6_): δ = 14.07 (butyl-C), 19.98 (butyl-C), 30.95(−CH_2_−), 42.30 (pyrazole-C), 44.71 (butyl-C), 46.12 (butyl-C), 59.83 (pyrazole-C), 124.37 (phenyl-C), 127.40 (phenyl-C), 127.56 (phenyl-C), 129.27 (phenyl-C), 130.97 (phenyl-C), 131.19 (phenyl-C), 133.96 (phenyl-C), 149.48 (phenyl-C), 155.69 (pyrazole-C), 165.35 (thiadiazole-C), 170.47 (C=O). HRMS (*m*/*z*): [M + H]^+^ calcd for C_23_H_24_N_6_O_3_S_2_, 497.1424; found, 497.1416.

2-((5-(Cyclohexylamino)-1,3,4-thiadiazol-2-yl)thio)-1-(5-(4-nitrophenyl)-3-phenyl-4,5-dihydro-1*H*-pyrazol-1-yl)ethan-1-one (**6f**): yield: 74%, mp: 136.0–137.4 °C. ^1^H-NMR (300 MHz, DMSO-*d*_6_): δ = 1.61 (6H, m, cyclohexyl), 1.92 (5H, d, *J* = 11.23 Hz, cyclohexyl), 3.23 (1H, dd, *J*_1_ = 5.07 Hz, *J*_2_ = 18.37 Hz, pyrazole), 3.90 (1H, dd, *J*_1_ = 19.60 Hz, *J*_2_ = 39.08 Hz, pyrazole), 4.40 (2H, m, −CH_2_), 5.73 (1H, dd, *J*_1_ = 5.07 Hz, *J*_2_ = 11.92 Hz, pyrazole), 7.46 (3H, m, monosubstituted benzene), 7.54 (2H, d, *J* = 8.83 Hz, disubstituted benzene), 7.77 (1H, s, seconder amine), 7.82 (2H, m, monosubstituted benzene), 8.19 (2H, d, *J* = 8.83 Hz, disubstituted benzene). ^13^C-NMR (75 MHz, DMSO-*d*_6_): δ = 9.03 (cyclohexyl-C), 24.67 (cyclohexyl-C), 25.66 (cyclohexyl-C), 32.46 (−CH_2_−), 37.23 (pyrazole-C), 45.82 (cyclohexyl-C), 53.91 (pyrazole-C), 124.35 (phenyl-C), 127.55 (phenyl-C), 129.27 (phenyl-C), 130.94 (phenyl-C), 131.18 (phenyl-C), 147.17 (phenyl-C), 148.68 (phenyl-C), 149.48 (phenyl-C), 155.66 (pyrazole-C), 165.36 (thiadiazole-C), 169.01, 169.45 (thiadiazole-C), 170.08 (C=O). HRMS (*m*/*z*): [M + H]^+^ calcd for C_25_H_26_N_6_O_3_S_2_, 523.1581; found, 523.1561.

1-(5-(4-Nitrophenyl)-3-phenyl-4,5-dihydro-1*H*-pyrazol-1-yl)-2-((5-(phenylamino)-1,3,4-thiadiazol-2-yl)thio)ethan-1-one (**6g**): yield: 86%, mp: 225.9–226.8 °C. ^1^H-NMR (300 MHz, DMSO-*d*_6_): δ = 3.25 (1H, m, pyrazole), 4.03 (1H, dd, *J*_1_ = 11.99 Hz, *J*_2_ = 18.36 Hz, pyrazole), 4.62 (2H, m, −CH_2_), 5.82 (1H, dd, *J*_1_ = 5.00 Hz, *J*_2_ = 11.90 Hz, pyrazole), 7.06 (1H, t, *J* = 7.35 Hz, monosubstituted benzene), 7.39 (2H, m, monosubstituted benzene), 7.52 (3H, m, monosubstituted benzene), 7.54 (2H, d, *J* = 8.86 Hz, monosubstituted benzene), 7.64 (4H, m, monosubstituted benzene), 7.85 (2H, d, *J* = 7.83 Hz, disubstituted benzene), 8.25 (2H, d, *J* = 8.83 Hz, disubstituted benzene), 10.44 (1H, s, seconder amine). ^13^C-NMR (75 MHz, DMSO-*d*_6_): δ = 37.01 (−CH_2_−), 42.33 (pyrazole-C), 59.92 (pyrazole-C), 117.80 (phenyl-C), 122.47 (phenyl-C), 124.36 (phenyl-C), 127.41 (phenyl-C), 127.56 (phenyl-C), 129.27 (phenyl-C), 129.56 (phenyl-C), 130.92 (phenyl-C), 131.22 (phenyl-C), 140.78 (phenyl-C), 147.20 (phenyl-C), 149.45 (phenyl-C), 152.41 (thiadiazole-C), 155.85 (pyrazole-C), 165.14 (thiadiazole-C), 165.60 (C=O). HRMS (*m*/*z*): [M + H]^+^ calcd for C_25_H_20_N_6_O_3_S_2_, 517.1111; found, 517.1109.

1-(5-(4-Nitrophenyl)-3-phenyl-4,5-dihydro-1*H*-pyrazol-1-yl)-2-((5-(*p*-tolylamino)-1,3,4-thiadiazol-2-yl)thio)ethan-1-one (**6h**): yield: 83%, mp: 184.5–186.7 °C. ^1^H-NMR (300 MHz, DMSO-*d*_6_): δ = 2.25 (3H, s, −CH_3_), 3.25 (1H, dd, *J*_1_ = 5.09 Hz, *J*_2_ = 18.33 Hz, pyrazole), 3.97 (1H, dd, *J*_1_ = 12.34 Hz, *J*_2_ = 18.07 Hz, pyrazole), 4.54 (2H, m, −CH_2_), 5.75 (1H, dd, *J*_1_ = 5.01 Hz, *J*_2_ = 11.92 Hz, pyrazole), 7.13 (2H, d, *J* = 8.35 Hz, disubstituted benzene), 7.41 (3H, m, monosubstituted benzene), 7.48 (2H, d, *J* = 1.50 Hz, disubstituted benzene), 7.55 (2H, m, monosubstituted benzene), 7.79 (2H, d, *J* = 7.86 Hz, disubstituted benzene), 8.19 (2H, d, *J* = 8.80 Hz, disubstituted benzene), 10.30 (1H, s, seconder amine). ^13^C-NMR (75 MHz, DMSO-*d*_6_): δ = 20.85 (−CH_3_), 37.04 (−CH_2_−), 42.33 (pyrazole-C), 59.91 (pyrazole-C), 117.94 (phenyl-C), 124.37 (phenyl-C), 127.41 (phenyl-C), 127.56 (phenyl-C), 129.28 (phenyl-C), 129.95 (phenyl-C), 130.92 (phenyl-C), 131.23 (phenyl-C), 131.44 (phenyl-C), 138.42 (phenyl-C), 147.19 (phenyl-C), 149.46 (phenyl-C), 151.89 (thiadiazole-C), 155.84 (pyrazole-C), 165.15 (thiadiazole-C), 165.84 (C=O). HRMS (*m*/*z*): [M + H]^+^ calcd for C_26_H_22_N_6_O_3_S_2_, 531.1268; found, 531.1251.

2-((5-((4-Methoxyphenyl)amino)-1,3,4-thiadiazol-2-yl)thio)-1-(5-(4-nitrophenyl)-3-phenyl-4,5-dihydro-1*H*-pyrazol-1-yl)ethan-1-one (**6i**): yield: 88%, mp: 180.9–181.6 °C. ^1^H-NMR (300 MHz, DMSO-*d*_6_): δ = 3.25 (1H, m, pyrazole), 3.79 (3H, s, −CH_2_), 4.03 (1H, dd, *J*_1_ = 12.05 Hz, *J*_2_ = 18.36 Hz, pyrazole), 4.58 (2H, m, −CH_2_), 5.81 (1H, dd, *J*_1_ = 5.01 Hz, *J*_2_ = 11.95 Hz, pyrazole), 6.97 (2H, d, *J* = 9.07 Hz, disubstituted benzene), 7.48 (2H, m, disubstituted benzene), 7.53 (3H, m, monosubstituted benzene), 7.61 (2H, m, monosubstituted benzene), 7.85 (2H, d, *J* = 7.83 Hz, disubstituted benzene), 8.25 (2H, d, *J* = 8.80 Hz, disubstituted benzene), 10.24 (1H, s, seconder amine). ^13^C-NMR (75 MHz, DMSO-*d*_6_): δ = 37.08 (−CH_2_−), 42.32 (pyrazole-C), 55.70 (methoxy-C), 59.91 (pyrazole-C), 114.74 (phenyl-C), 119.68 (phenyl-C), 124.37 (phenyl-C), 127.41 (phenyl-C), 127.56 (phenyl-C), 129.28 (phenyl-C), 130.93 (phenyl-C), 131.22 (phenyl-C), 134.28 (phenyl-C), 147.20 (phenyl-C), 149.45 (phenyl-C), 151.35 (phenyl-C), 155.08 (thiadiazole-C), 155.83 (pyrazole-C), 165.19 (thiadiazole-C), 166.34 (C=O). HRMS (*m*/*z*): [M + H]^+^ calcd for C_26_H_22_N_6_O_4_S_2_, 547.1217; found, 547.1220.

2-((5-((4-Chlorophenyl)amino)-1,3,4-thiadiazol-2-yl)thio)-1-(5-(4-nitrophenyl)-3-phenyl-4,5-dihydro-1*H*-pyrazol-1-yl)ethan-1-one (**6j**): yield: 88%, mp: 162.5–163.4 °C. ^1^H-NMR (300 MHz, DMSO-*d*_6_): δ = 3.23 (1H, m, pyrazole), 3.96 (1H, dd, *J*_1_ = 11.99 Hz, *J*_2_ = 18.42 Hz, pyrazole), 4.75 (2H, m, −CH_2_), 5.74 (1H, dd, *J*_1_ = 4.80 Hz, *J*_2_ = 11.80 Hz, pyrazole), 6.97 (2H, d, *J* = 9.07 Hz, disubstituted benzene), 7.48 (2H, m, disubstituted benzene), 7.53 (3H, m, monosubstituted benzene), 7.61 (2H, m, monosubstituted benzene), 7.85 (2H, d, *J* = 7.83 Hz, disubstituted benzene), 8.25 (2H, d, *J* = 8.80 Hz, disubstituted benzene), 10.52 (1H, s, seconder amine). ^13^C-NMR (75 MHz, DMSO-*d*_6_): δ = 37.00 (−CH_2_−), 42.87 (pyrazole-C), 60.02 (pyrazole-C), 119.33 (phenyl-C), 124.36 (phenyl-C), 124.47 (phenyl-C), 125.82 (phenyl-C), 127.48 (phenyl-C), 127.62 (phenyl-C), 129.29 (phenyl-C), 129.36 (phenyl-C), 130.88 (phenyl-C), 131.32 (phenyl-C), 139.66 (phenyl-C), 147.29 (phenyl-C), 149.33 (phenyl-C), 149.44 (thiadiazole-C), 155.86 (pyrazole-C), 165.10 (thiadiazole-C), 165.23(C=O). HRMS (*m*/*z*): [M + H]^+^ calcd for C_25_H_19_N_6_O_3_S_2_Cl, 551.0721; found, 551.0723.

### 2.2 Biological Activity Studies

#### 2.2.1 Cytotoxicity Test

The principle of the MTT test used to determine the metabolic activity of living cells is based on the reduction of colorless 3-(4,5-dimethylthiazol-2-yl)-2,5 diphenyltetrazolium salt to the purple-colored formazan product. Thanks to this color change, the cell viability rate can be determined spectrometrically.^33^Table 1 presents the results of the MTT experiment performed with the 24 h procedure using two cancer lines (A549 and MCF-7) and a healthy cell line (NIH3T3). Doxorubicin is used as a reference drug. MTT assays were performed as previously described.^34−36^

**Table 1:** IC_50_ (μM) Values of Synthesized Compounds (**6a–6j**)^a^

compounds	A549	MCF-7	NIH3T3	SI for A549	SI for MCF-7
**6a**	13.90 ± 0.25	>100	86.68 ± 4.07	6.24	<0.87
**6b**	15.80 ± 0.75	63.40 ± 2.09	47.22 ± 4.53	0.74	2.99
**6c**	27.92 ± 1.28	>100	33.19 ± 2.37	1.19	<0.33
**6d**	5.18 ± 0.16	35.72 ± 2.24	11.91 ± 0.49	2.30	0.33
**6e**	>100	>100	41.98 ± 4.19	<0.42	<0.42
**6f**	>100	>100	60.50 ± 3.72	<0.61	<0.61
**6g**	1.54 ± 0.10	15.93 ± 0.05	11.12 ± 0.21	7.22	0.70
**6h**	29.05 ± 1.01	92.64 ± 1.89	>100	>3.44	>1.08
**6i**	16.64 ± 0.21	>100	>100	>6.01	
**6j**	8.49 ± 0.67	67.25 ± 3.63	48.26 ± 1.72	5.68	0.72
**doxorubicin**	2.667 ± 0.12	1.589 ± 0.10	>1000	>374.95	>629.33

a The test results were expressed as means of quartet assays.

#### 2.2.2 Analysis of Mitochondrial Membrane Potential by Flow Cytometry

The BD MitoScreen Mitochondrial Membrane Potential Detection JC-1 Kit (available from ref (37)) was used for the mitochondrial membrane potential (MMP) test. In the first step, A549 cells seeded in 25 mL flasks were incubated for 24 h in a 5% CO_2_ incubator. At the end of the 24 h incubation period, the medium in the flasks was removed, and compound **6g** and the reference drug were added to the flasks (at IC_50_ concentration). Cells were collected and centrifuged in line with the instructions in the kit at the conclusion of this period. The upper part was removed, JC-1 dye was added, and the mixture was incubated for 10–15 min at 37 °C. The CytoFLEX flow cytometer (Beckman Coulter Life Sciences, USA) and CytExpert for CytoFLEX Acquisition and Analysis Software Version 2.2.0.97 were used to read it after two washings with washing solution at the conclusion of the period.

#### 2.2.3 EGFR Inhibition Assay

The EGFR Kinase Assay Kit (available from ref (38)) was used for the EGFR inhibition. The kit protocol was followed, and the experiment was carried out in vitro.

### 2.3 Molecular Docking Studies

Molecular docking studies were performed using an in silico procedure to define the binding modes of active compound **6g** in the active regions of the crystal structures of EGFR (PDB ID: 1M17),^39^ which were retrieved from the Protein Data Bank server (www.pdb.org). The *Schrödinger Maestro*(40) interface was used to construct the enzyme structures, which were subsequently submitted to the *Protein Preparation Wizard* protocol of the *Schrödinger Suite 2020*. The LigPrep module^41^ was used to properly assign the protonation states and atom types to the ligand during preparation. The structures were given bond order assignments and hydrogen atoms. The *Glide* module^42^ was used to generate the grid, and the standard precision (SP) docking mode was used for docking runs.

### 2.4 Molecular Dynamics Simulation Studies

Molecular dynamics (MD) simulations, which are considered an important computational tool to evaluate the time-dependent stability of a ligand at an active site for a drug–receptor complex, were performed for compound **6g** within the scope of this study. MD studies were performed for 100 ns as previously reported.^43−46^ Following the completion of the system setup, the MD simulation was run using the settings. The Desmond application calculated the values for *R*_g_ (radius of gyration), root mean square fluctuation (RMSF), and root mean square deviation (RMSD).^47,48^

## 3 Results and Discussion

### 3.1 Chemistry

**Scheme 1:**
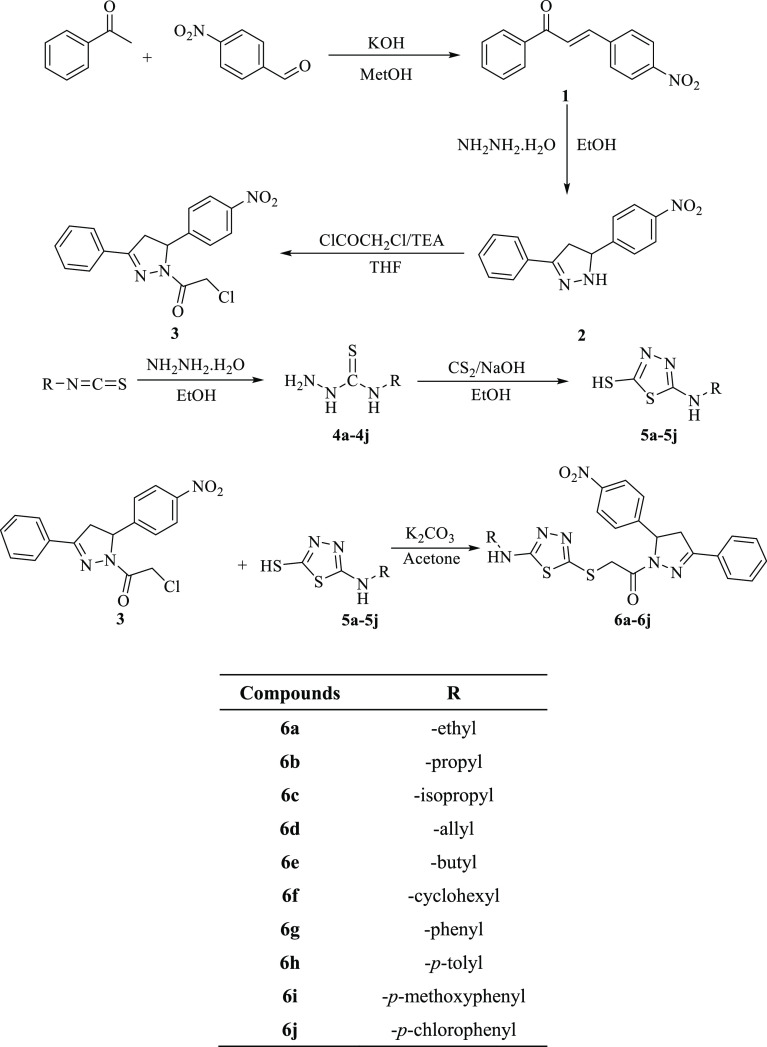
Synthetic Route of Compounds **6a–6j**

Target compounds (**6a–6j**) were prepared in a total of six steps. The synthetic route to the obtained compounds **6a–6j** is presented in Scheme 1. In the first step, 3-(4-nitrophenyl)-1-phenylprop-2-en-1-one (**1**) was obtained from acetophenone and 4-nitrobenzaldehyde using Claisen–Schmidt reaction. Compound **1** was refluxed with hydrazine hydrate, and the ring closure reaction was performed to obtain 5-(4-nitrophenyl)-3-phenyl-4,5-dihydro-1*H*-pyrazole (**2**).

Compound **2** was acetylated using an ice bath. Compounds **4a–4j** were obtained with the reaction between isothiocyanates and hydrazine hydrate. The thiadiazol derivatives (**5a–5j**) were gained using a ring closure reaction in basic conditions. The thiadiazoles (**5a–5j**) obtained because of this were reacted with compound **3** to obtain target compounds (**6a–6j**). The structures of the compounds obtained were established by spectroscopic methods, namely, ^1^H-NMR, ^13^C-NMR, and HRMS (Supporting Information).

Characteristic peaks of the protons belonging to the pyrazoline ring, ethylene group, and secondary amine were observed in ^1^H-NMR results. The peaks of the pyrazoline were obtained in the range of 3.17–3.32, 3.90–4.03, and 5.73–5.82 ppm. The characteristic signals of the ethylene −CH_2_ protons were found at 4.40–4.78 ppm. In the secondary amine peaks, if the bonding group is aliphatic, the peaks in the range of 7.72–7.85 ppm were obtained, but if the bonded group is aromatic, the peaks in the range of 10.24–10.52 ppm were obtained. In ^13^C-NMR analyses, the peaks of carbon atoms with carbonyl groups were consistently around 165 ppm.

### 3.2 Evaluation of the Biological Activity Studies

#### 3.2.1 Cytotoxicity Test

Cytotoxicity assays of compounds **6a–6j** were performed with the 24 h MTT procedure. The results are presented in Table 1. There are three compounds that show activity against the A549 cell line with an IC_50_ < 10 μM. These compounds include allyl (**6d**), phenyl (**6g**), and 4-chlorophenyl (**6j**) moieties. In contrast, no activity was observed in the compounds containing isobutyl (**6e**) and cyclohexyl (**6f**) moieties. Compound **6d** showed activity against the A549 cell line with an IC_50_ = 5.176 ± 0.164 μM. Compound **6g** showed activity against the A549 cell line with an IC_50_ = 1.537 ± 0.097 μM. Compound **6j** showed activity against the A549 cell line with an IC_50_ = 8.493 ± 0.667 μM. Against the MCF-7 cell line, all compounds showed less activity than IC_50_ = 10 μM. But compounds **6d**, **6g**, and **6j** were the most active compounds with their IC_50_ = 35.724 ± 2.237 μM, IC_50_ = 15.925 ± 0.054 μM, and IC_50_ = 67.246 ± 3.627 μM activity values against the MCF-7 cell line, respectively. In this case, it is possible to say that the compounds exhibited a selective activity against the A549 cell line. Compound **6d** showed cytotoxicity with an IC_50_ = 11.907 ± 0.486 μM. When the healthy cell line was examined, compound **6g** showed cytotoxicity with an IC_50_ = 11.115 ± 0.205 μM. Compound **6j** showed cytotoxicity with an IC_50_ = 48.260 ± 1.717 μM. The selectivity indexes of compounds **6d**, **6g**, and **6j** were calculated as 2.3, 7.23, and 5.68, respectively. In this case, compound **6g** was both the most effective derivative against the A549 cell line and the derivative with the highest selectivity index. It can be predicted that the side effect and toxic effect profile of compound **6g** will be less than that of compounds **6d** and **6j**.

#### 3.2.2 Analysis of MMP by Flow Cytometry

**Figure 2:**
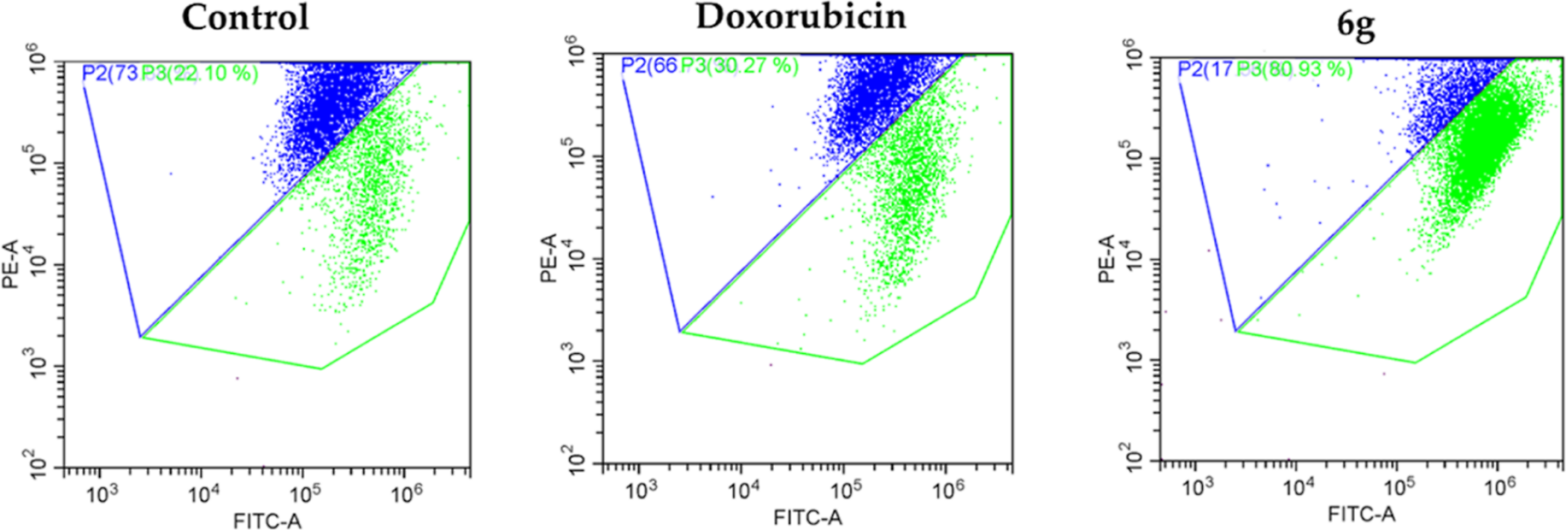
Analysis of mitochondrial membrane potential of compound **6g** and doxorubicin.

The membrane potential of mitochondria, which have a central role in apoptosis, is a measurable change in flow cytometry.^49^ Mitochondria are an important target in the case of cancer cells that are resistant to drug therapy. Mitochondria play a key regulatory role in the apoptotic pathway. Therefore, they are identified as an important target for cancer therapy.^50^ Mitochondria generate a membrane potential using oxidizable substrates. The provision of these substrates is associated with the growth factor. Therefore, growth factor reduction or extracellular glucose loss reduces the MMP. If growth factor or glucose deprivation persists, the cells ultimately undergo apoptosis that is initiated by cytochrome *c* release from mitochondria.^51^ For this purpose, the mitochondrial membrane potential of compound **6g** (the most active compound) was determined against the A549 cell line by in vitro flow cytometric methods. Both the inhibitory compound and the reference drug were administered at IC_50_ concentrations. After a 24 h incubation period (5% CO_2_), mitochondrial membrane potential was determined by flow cytometry using JC-1 dye. The resulting flow cytometry diagrams are presented in Figure 2. According to the results obtained (Figure 2), while doxorubicin showed 30.27% mitochondrial membrane potential, compound **6g** showed 80.93% mitochondrial membrane potential. This rate is promising. The high MMP potential of compound **6g** provides both the restriction of resistance development and a safer treatment with the apoptotic pathway.

#### 3.2.3 EGFR Inhibition Assay

EGFR kinase, which belongs to the ErbB family of tyrosine kinases, is known to regulate the signaling pathways of cell survival, migration, proliferation, differentiation, and adhesion.^52^ Inhibition of this member of the tyrosine kinase family, which is a new approach in cancer chemotherapy, is important. Therefore, the EGFR inhibition potential of compound **6g**, which is the most active compound, was evaluated by the in vitro kit method. The IC_50_ value for compound **6g** was calculated using the kit procedure. According to the results obtained, compound **6g** shows inhibitory activity on the EGFR enzyme with a value of IC_50_ = 0.024 ± 0.002 μM. For Erlotinib, the IC_50_ value was found to be 0.002 ± 0.001 μM.

### 3.3 Molecular Docking Studies

**Figure 3:**
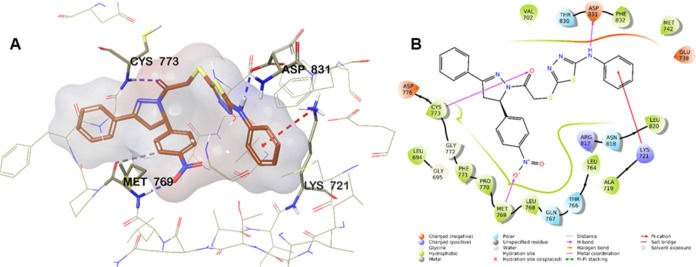
(A) 2D interaction of compound **6g** at the binding region (PBDID: 1M17). (B) Three-dimensional interacting mode of compound **6g** in the active region of EGFR enzyme (PDB ID: 1M17). Compound **6g** is colored orange; the active site is colored beige.

As a result of the activity studies, it was seen that compound **6g** had the highest activity among the synthesized compounds. Docking studies were carried out to examine the binding modes of compound **6g** with the enzyme active site. The docking studies were performed, with the EGFR enzyme active site. Crystal structure of the EGFR enzyme (PDB code: 1M17)^39^ was retrieved from the PDB data bank. In the studies, the docking technique was performed with the Glide 7.1.^42^Figure 3 presents the obtained docking poses.

Figure 3A presents a 2D view, while Figure 3B presents a 3D view of the interaction of compound **6g** with the EGFR enzyme active site. The benzene ring near the thiadiazole ring formed a cation−π interaction with the amine of Lys721. The amine group between the benzene and thiadiazole rings established a hydrogen bond with the carbonyl of Asp831. Another hydrogen bond was noted between the carbonyl near the pyrazole ring and the amine of Cys773. The last hydrogen bond was detected between the nitro moiety and the amine of Met769. Also, the benzene ring to which this nitro moiety was attached formed an aromatic hydrogen bond with the carbonyl of Met769.

### 3.4 MD Simulation Studies

The MD simulation method is commonly used to investigate the dynamic behavior of proteins or protein–ligand complexes. In the current study, to evaluate the stability of the docking complex formed between the promising molecule **6g** and EGFR (PDB ID: 1M17), were taken into consideration for the 100 ns MD simulation study in an explicit hydration environment.

**Figure 4:**
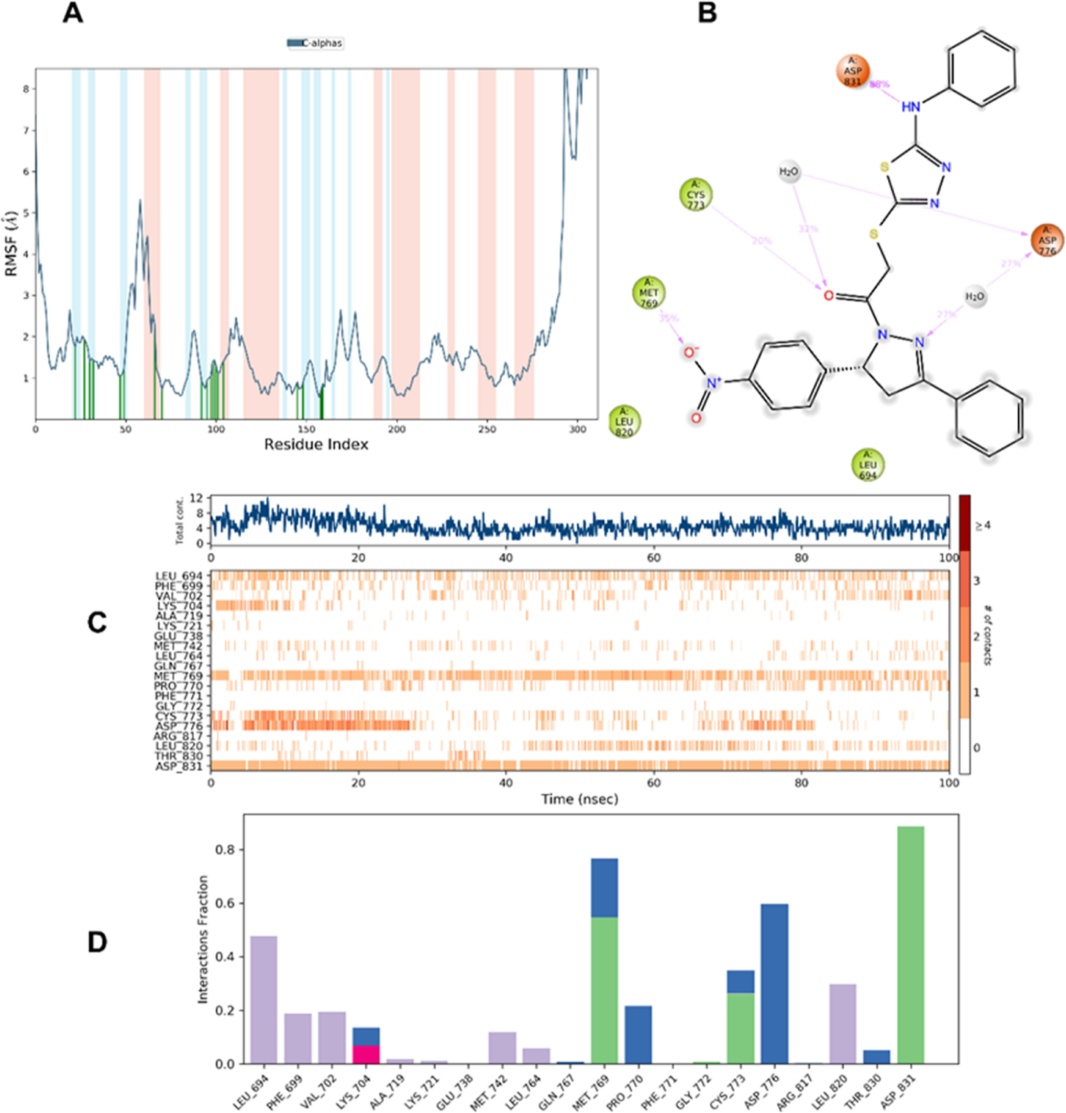
MD simulation analysis of compound **6g** in complex EGFR enzyme (PDB ID: 1M17) (A) protein RMSF, (B) 2D interaction diagram, and (C,D) protein–ligand contact analysis of MD trajectory.

The results for the compound **6g**–EGFR enzyme complex are shown in Figure 4. Certain amino acids are known to play a significant role in the protein–ligand complex’s stability during the MD simulation investigation. Over the duration of the simulation period, the individual residue variation and conformational changes across the protein chain may be observed by looking at the RMSF parameter (Figure 4A). The suggested chemical binds strongly to the active site of the EGFR protein if the atoms in the active site and main chain are minimally displaced, indicating that the conformational change is minimal. In the RMSF graphic, α-helix areas are shown by red, β-banded regions are represented by blue, and the loop region is represented by white. Compared to the protein’s loop region, the α-helical and β-helical sections are stiffer. Moreover, there are fewer fluctuations here. Vertical green lines on the plot’s *X*-axis show how contacting residues between each protein chain and its ligand contributed to the overall result.

As per the RMSF plot, compound **6g** contacted 20 amino acids of EGFR protein, namely, Leu694, Phe699, Val702, Lys704, Val718, Lys721, Glu738, Met742, Leu764, Gln767, Met769, Pro770, Phe771, Gly772, Cys773, Asp776, Arg817, Leu820, Thr830, and Asp831. Major fluctuations were observed in the C-terminal and loop regions, which are positioned away from the binding pocket. On the other hand, in the 1M17–**6g** complex, the fluctuations of the simulated compound have no substantial changes.

By watching the MD simulation video, aromatic hydrogen bonds were determined for 100 ns s. Accordingly, the aromatic hydrogen bonds formed can be listed as follows: between the 4-nitrophenyl ring of compound **6g** and the carbonyl of Met769; between the phenyl ring near the pyrazole ring of compound **6g** and the carbonyl of Leu694; between the phenyl ring near the secondary amine group of compound **6g** and the carbonyl of Ala719; between the phenyl ring near the secondary amine group of compound **6g** and the carbonyl of Ile720; between the phenyl ring near the secondary amine group of compound **6g** and the carbonyl of Leu764; between the phenyl ring near the secondary amine group of compound **6g** and the carbonyl of Asp831; between the phenyl ring near to the secondary amine group of compound **6g** and the hydroxyl of Thr766; and between the phenyl ring near the secondary amine group of compound **6g** and the hydroxyl of Thr830.

As seen in Figure 4B, 88% interaction was achieved between the secondary amine group and Asp831. The nitro group in the structure interacted with Met769 at a rate of 35%. These findings show that these interactions are very essential for stability. Again, a 20% interaction was obtained between the carbonyl moiety in the structure and Cys773. The nitrogen atom of the pyrazole ring interacted with Asp776 at a rate of 27%. Also, the results of protein–ligand contact analysis are seen in Figure 4C,D.

Consequently, it was seen that compound **6g** provides the stability in the enzyme active site of EGFR by interacting especially Met769, Asp831, Asp776, and Cys773. It was concluded by analyzing the MD simulation video where the phenyl ring near the pyrazole acts in a flexible conformation. It was thought that the placement of chemical structures of larger volume instead of the phenyl ring may contribute to the stability positively and therefore may obtain a stronger binding profile.

## 4 Conclusions

Today, EGFR inhibitors are used in the treatment of cancer types with high mortality rates such as lung cancer. New EGFR inhibitors are a great source of hope for the treatment of diseases such as lung cancer. Therefore, in this study, it was desired to synthesize new EGFR inhibitors with improved effects. It is expected that the newly designed compounds containing pyrazole and thiadiazole in their structures will reach high activity values through these structures they contain. After the six-step synthesis and characterization studies, activity studies were started. Cytotoxicity experiments showed that the most active compounds were **6d**, **6g**, and **6j**. These compounds had IC_50_ = 5.176 ± 0.164 μM, IC_50_ = 1.537 ± 0.097 μM, and IC_50_ = 8.493 ± 0.667 μM activity values against the A549 cell line, respectively. The compound with the highest activity, **6g**, showed 80.93% mitochondrial membrane potential. Compound **6g** shows an inhibitory activity on the EGFR enzyme with a value of IC_50_ = 0.024 ± 0.002 μM. Molecular docking and MD simulations showed that the compound fully fits into the **6g** EGFR active site.

## Supporting Information Available

^1^H-NMR, ^13^C-NMR, and HRMS spectrums of compounds **6a**–**6j** (PDF)
MD simulation video (AVI)

## Supplementary Material

ao3c04635_si_001.pdf

ao3c04635_si_002.avi
