# Supplementary material for: Synthesis and Anticancer Activities of Pyrazole–Thiadiazole-Based EGFR Inhibitors
Source: ACS Omega. 2023 Aug 17;8(34):31500–9. doi: 10.1021/acsomega.3c04635 (PMC10468883; doi:10.1021/acsomega.3c04635)
Supplement: Supplementary file 1 — ao3c04635_si_001.pdf [file ao3c04635_si_001.pdf]

Synthesis and anticancer activities of pyrazole-thiadiazole based EGFR inhibitors

Berkant Kurban [a,b], Begüm Nurpelin Sağlık [b,c]*, Derya Osmaniye [b,c], Serkan Levent [b,c], Yusuf Özkay [b,c] and Zafer Asım Kaplancıklı [b]

[a]*Department of Pharmaceutical Chemistry, Faculty of Pharmacy, Afyonkarahisar Health Sciences University, Afyonkarahisar, Turkey*

[b]*Department of Pharmaceutical Chemistry, Faculty of Pharmacy, Anadolu University, Eskişehir, Turkey*

[c]*Central Research Laboratory (MERLAB), Faculty of Pharmacy, Anadolu University, Eskişehir, Turkey*

* Corresponding author

E-mail address: bnsaglik@anadolu.edu.tr (B.N. Sağlık)

Tel: +90-222-3350580/3774, *Fax*: +90-222-3350750

Address: Anadolu University, Faculty of Pharmacy, Department of Pharmaceutical Chemistry, 26470, Eskişehir, Turkey

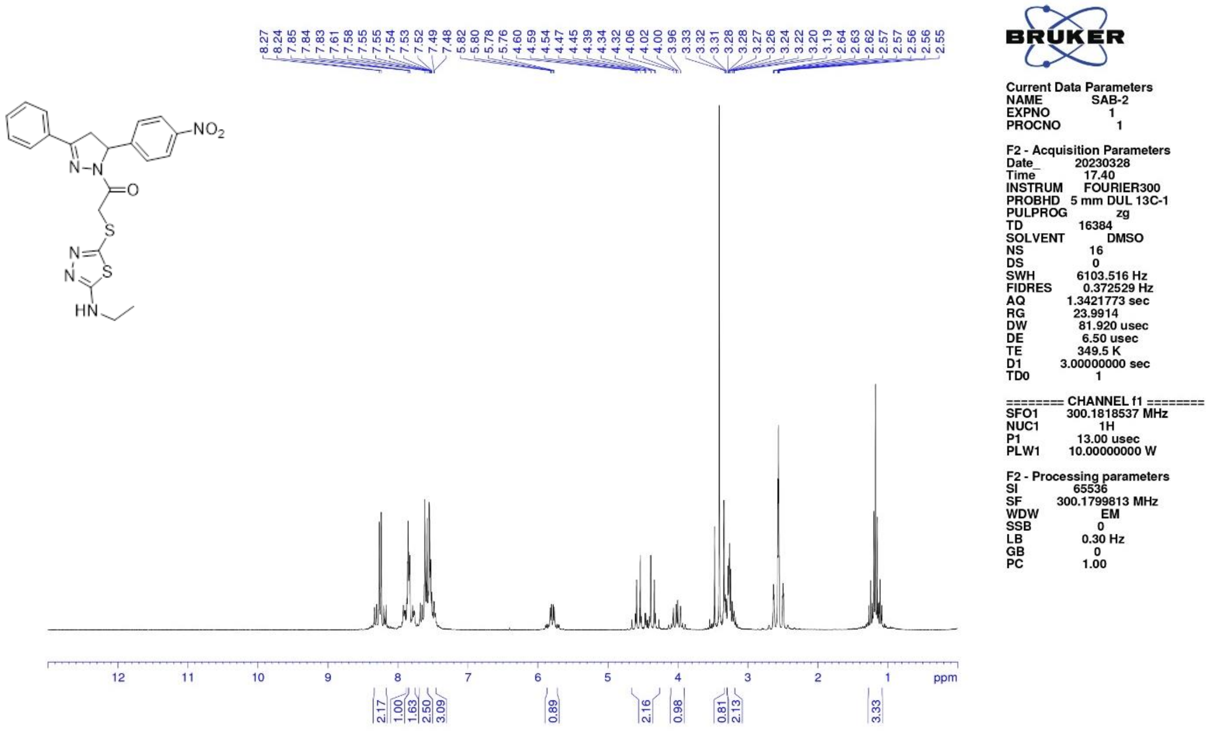

Spectra 1. ^{1}H-NMR spectra of compound **6a**

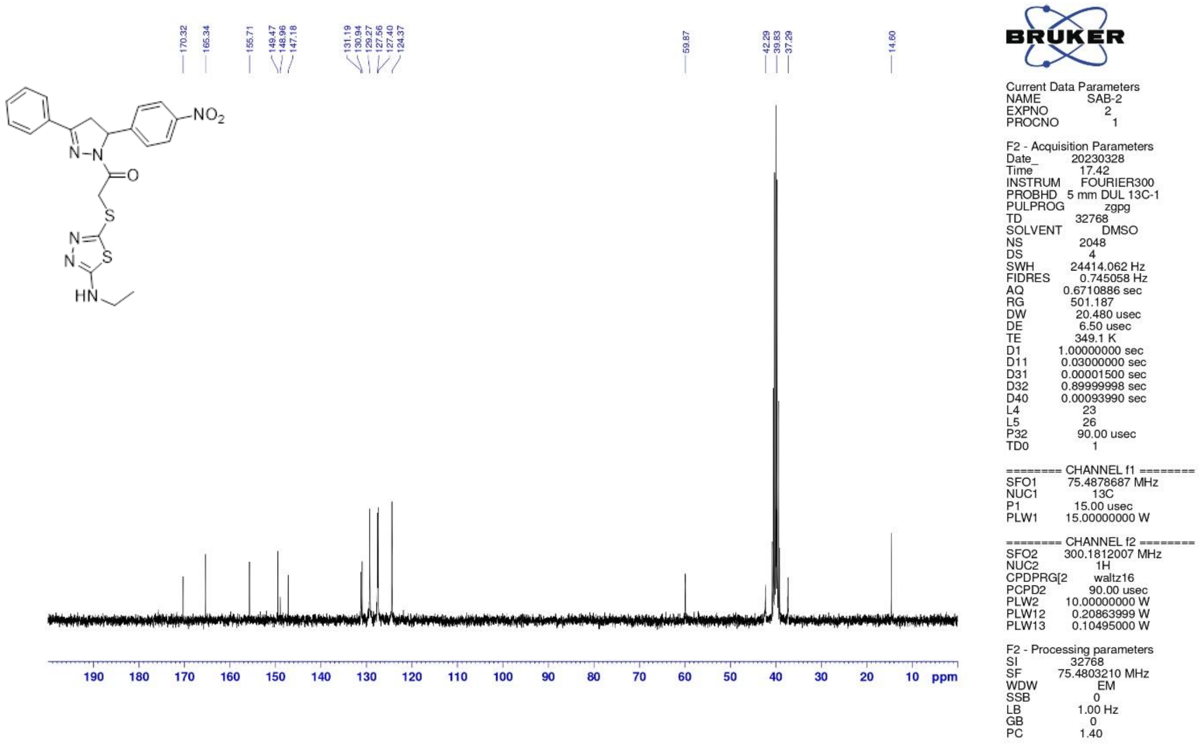

Spectra 2. ^{13}C-NMR spectra of compound **6a**

Data File: C:\LabSolutions\Data\Analiz\derya\SAB-2_184.lcd

Elmt	Val.	Min	Max	Elmt	Val.	Min	Max	Elmt	Val.	Min	Max	Elmt	Val.	Min	Max	Use Adduct
H	1	8	40	O	2	0	4	S	2	2	2	Ru	2	0	0	H
C	4	9	40	F	1	0	0	Cl	1	0	0	Pd	2	0	0	
N	3	2	6	P	3	0	0	Br	1	0	0	I	3	0	0	

Error Margin (ppm):	5	DBE Range:	5.0 - 25.0	Electron Ions:	both
HC Ratio:	unlimited	Apply N Rule:	yes	Use MSn Info:	yes
Max Isotopes:	3	Isotope RI (%):	1.00	Isotope Res:	9000
MSn Iso RI (%):	10.00	MSn Logic Mode:	AND	Max Results:	200

Event#: 1 MS(E+) Ret. Time : 3.160 Scan# : 475

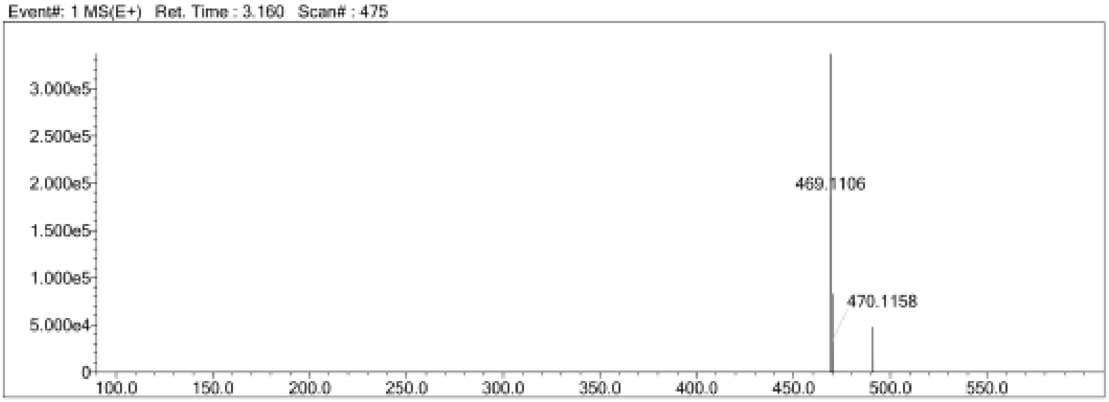

Measured region for 469.1106 m/z

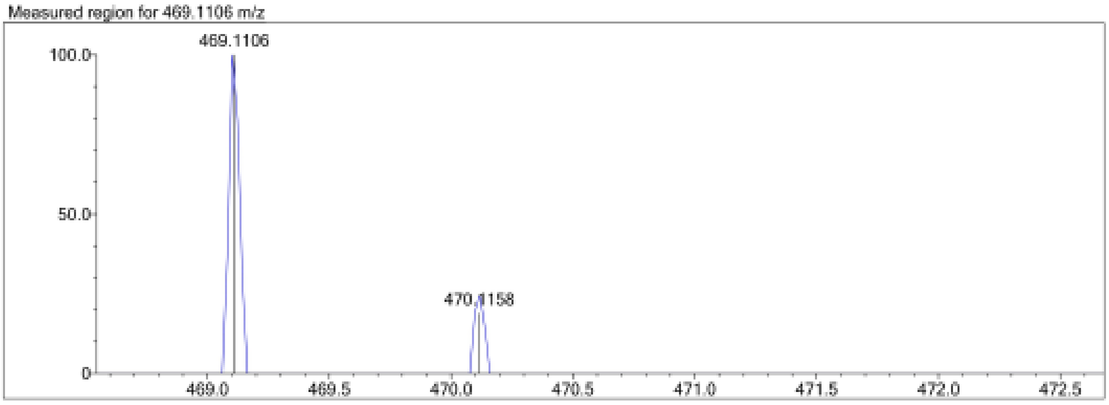

C21 H20 N6 O3 S2 [M+H]+ : Predicted region for 469.1111 m/z

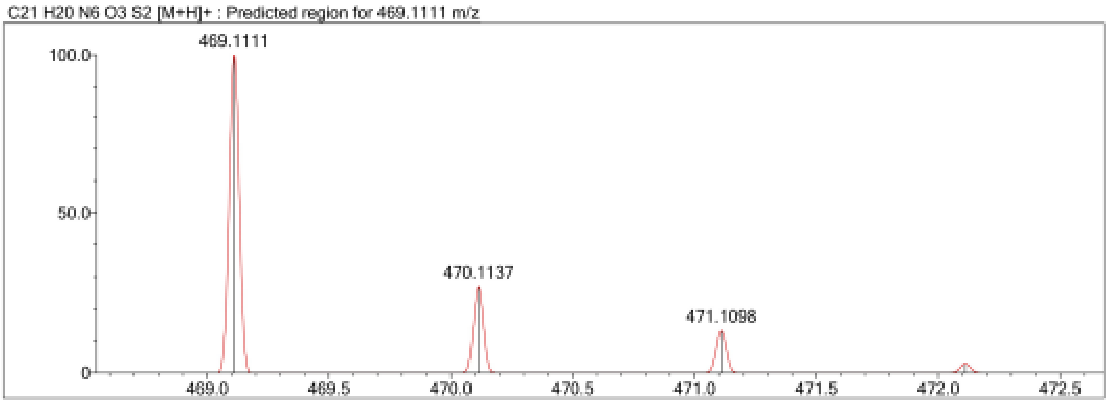

Rank	Score	Formula (M)	Ion	Meas. m/z	Pred. m/z	Df. (mDa)	Df. (ppm)	Iso	DBE
1	0.00	C21 H20 N6 O3 S2	[M+H]+	469.1106	469.1111	-0.5	-1.07	0.00	15.0

Spectra 3. HRMS spectra of compound **6a**

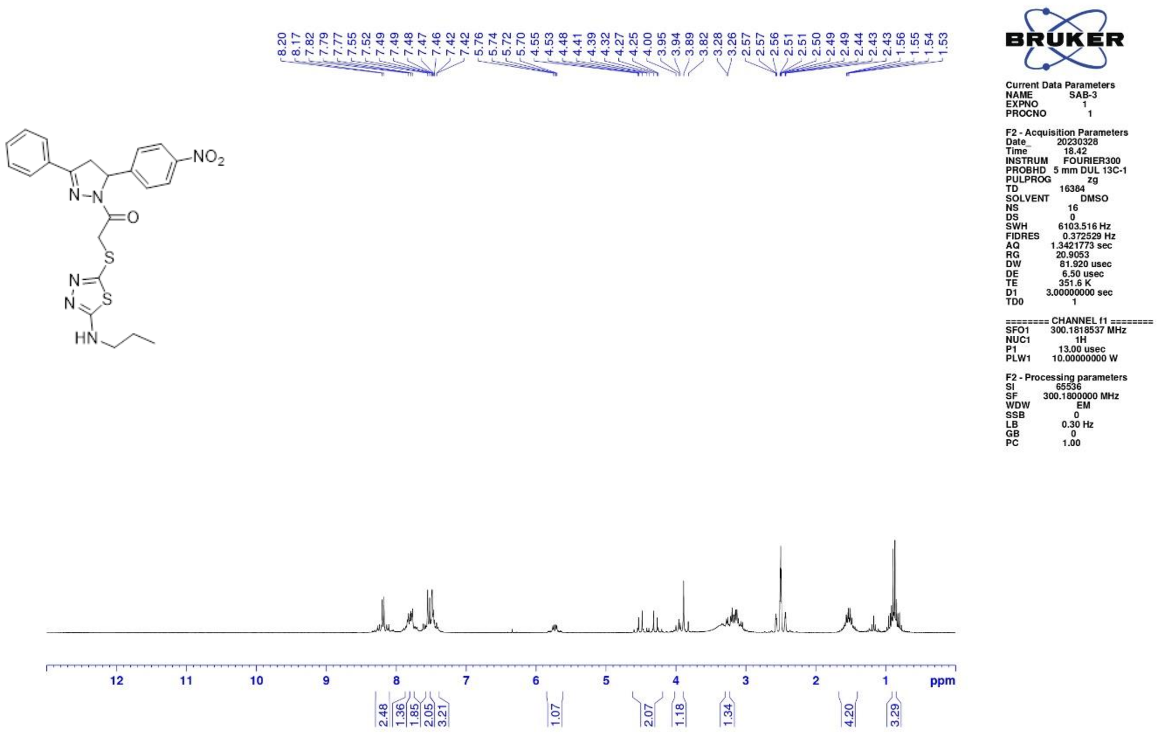

Spectra 4. ^{1}H-NMR spectra of compound **6b**

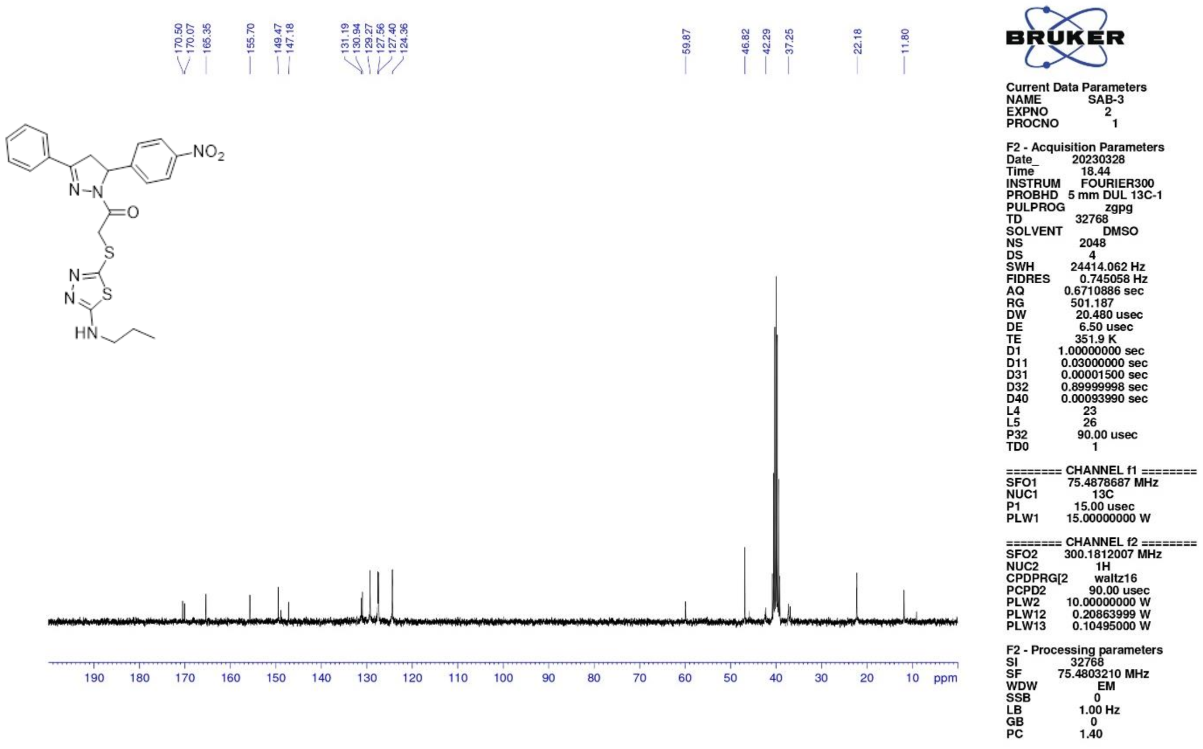

Spectra 5. ^{13}C-NMR spectra of compound **6b**

Data File: C:\LabSolutions\Data\Analiz\derya\SAB-3_185.lcd

Elmt	Val.	Min	Max	Elmt	Val.	Min	Max	Elmt	Val.	Min	Max	Elmt	Val.	Min	Max	Use Adduct
H	1	8	40	O	2	0	4	S	2	2	2	Ru	2	0	0	H
C	4	9	40	F	1	0	0	Cl	1	0	0	Pd	2	0	0	
N	3	2	6	P	3	0	0	Br	1	0	0	I	3	0	0	

Error Margin (ppm):	5	DBE Range:	5.0 - 25.0	Electron Ions:	both
HC Ratio:	unlimited	Apply N Rule:	yes	Use MSn Info:	yes
Max Isotopes:	3	Isotope RI (%):	1.00	Isotope Res:	9000
MSn Iso RI (%):	10.00	MSn Logic Mode:	AND	Max Results:	200

Event#: 1 MS(E+) Ret. Time : 3.573 Scan# : 537

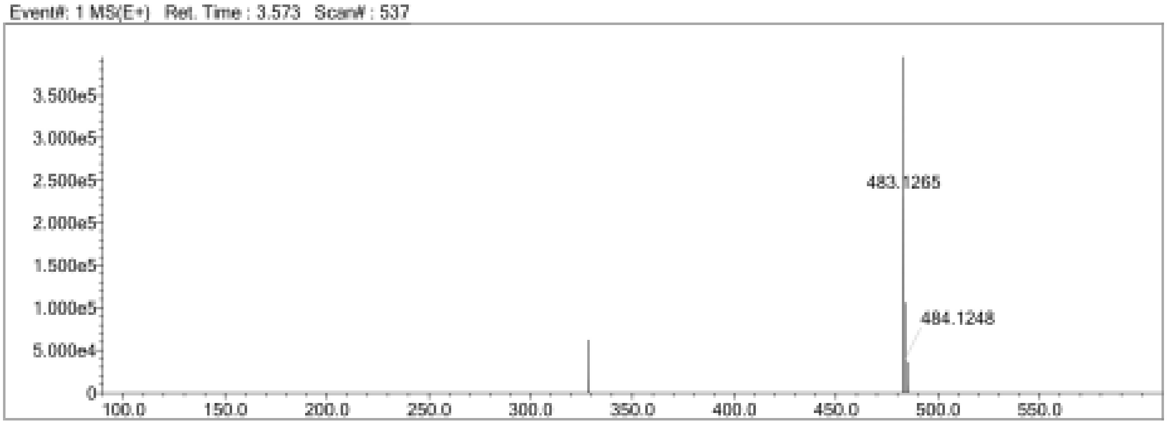

Measured region for 483.1265 m/z

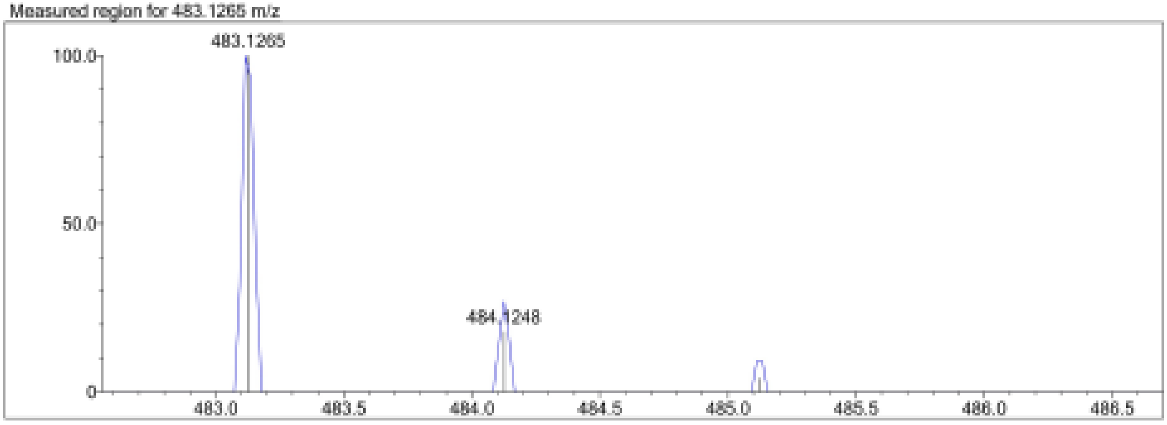

C22 H22 N6 O3 S2 [M+H]+ : Predicted region for 483.1268 m/z

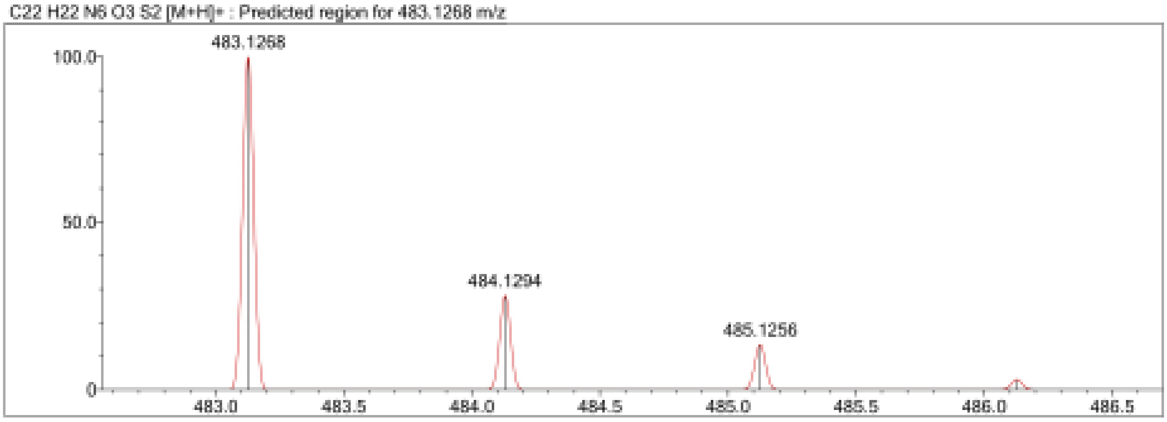

Rank	Score	Formula (M)	Ion	Meas. m/z	Pred. m/z	Df. (mDa)	Df. (ppm)	Iso	DBE
1	74.97	C22 H22 N6 O3 S2	[M+H]+	483.1265	483.1268	-0.3	-0.62	74.97	15.0

Spectra 6. HRMS spectra of compound **6b**

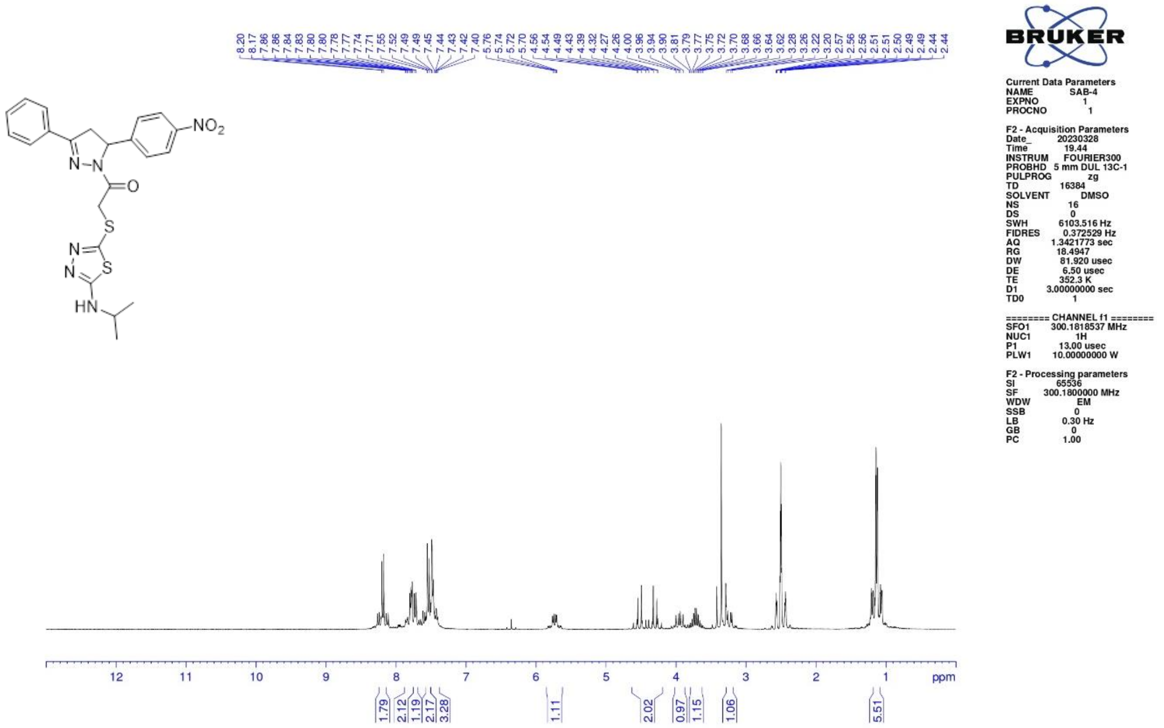

Spectra 7. ^{1}H-NMR spectra of compound **6c**

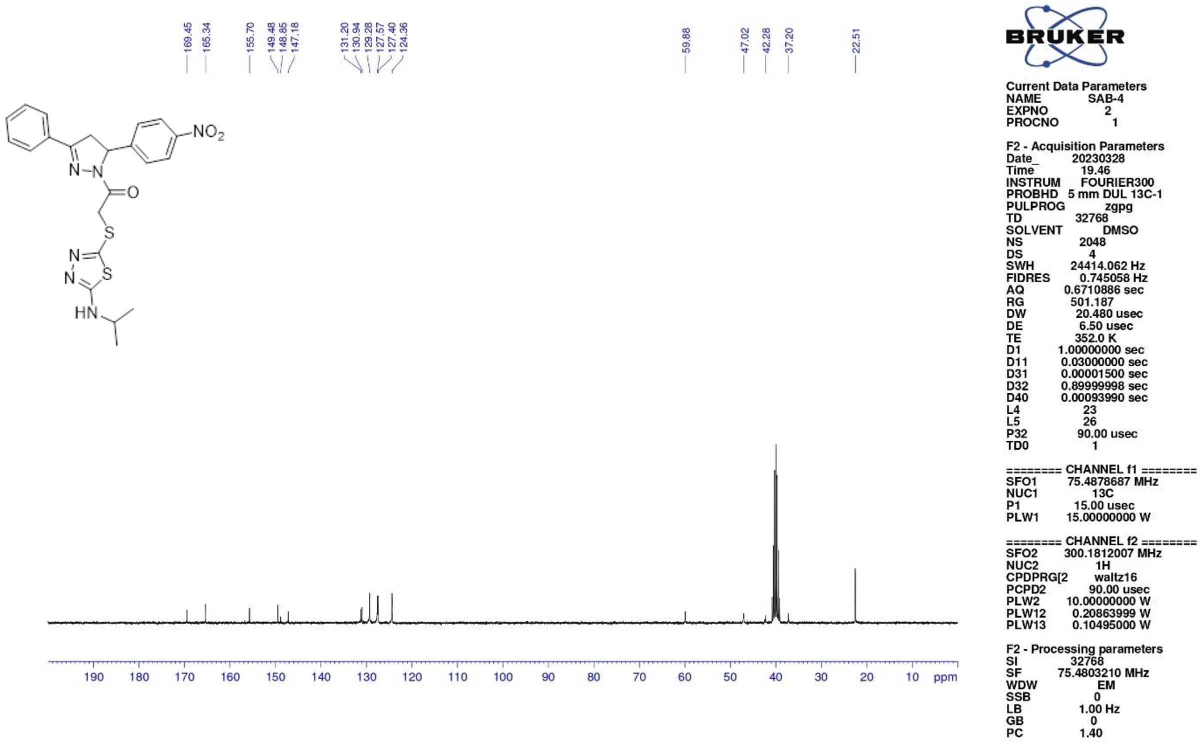

Spectra 8. ^{13}C-NMR spectra of compound **6c**

Data File: C:\LabSolutions\Data\Analiz\derya\SAB-4_186.lcd

Elmt	Val.	Min	Max	Elmt	Val.	Min	Max	Elmt	Val.	Min	Max	Elmt	Val.	Min	Max	Use Adduct
H	1	8	40	O	2	0	4	S	2	2	2	Ru	2	0	0	H
C	4	9	40	F	1	0	0	Cl	1	0	0	Pd	2	0	0	
N	3	2	6	P	3	0	0	Br	1	0	0	I	3	0	0	

Error Margin (ppm): 5
HC Ratio: unlimited
Max Isotopes: 3
MSn Iso RI (%): 10.00

DBE Range: 5.0 - 25.0
Apply N Rule: yes
Isotope RI (%): 1.00
MSn Logic Mode: AND

Electron Ions: both
Use MSn Info: yes
Isotope Res: 9000
Max Results: 200

Event#: 1 MS(E+) Ret. Time : 3.347 -> 3.347 Scan# : 503 -> 503

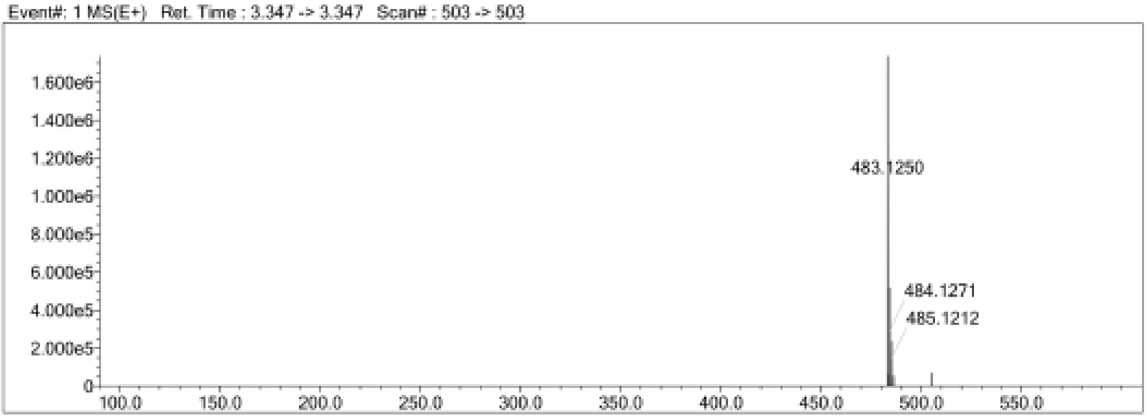

Measured region for 483.1250 m/z

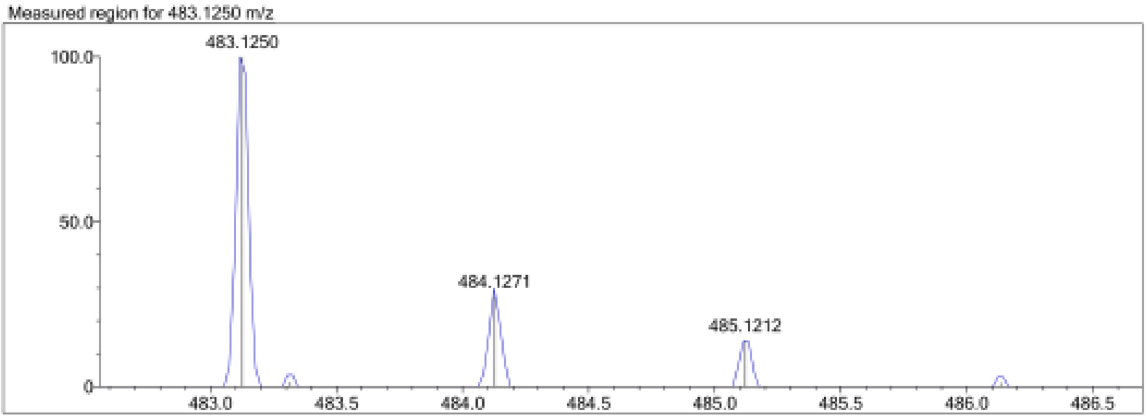

C22 H22 N6 O3 S2 [M+H]+ : Predicted region for 483.1268 m/z

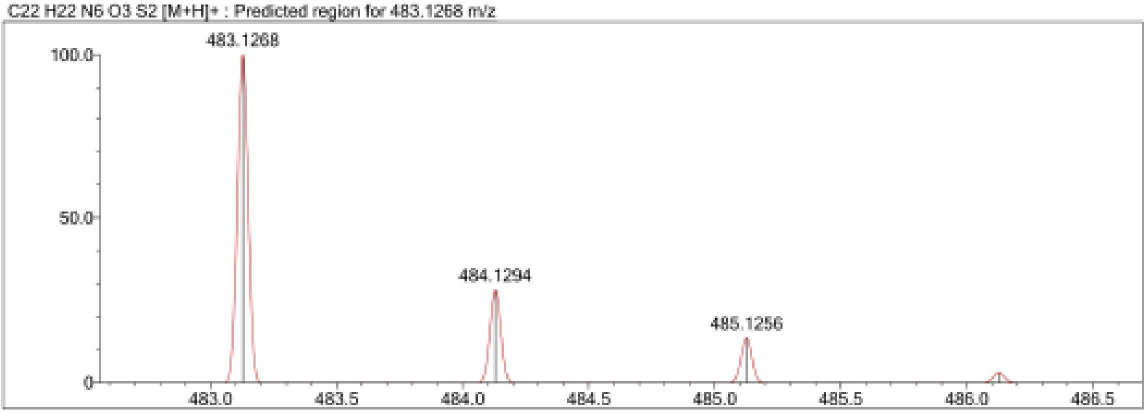

Rank	Score	Formula (M)	Ion	Meas. m/z	Pred. m/z	Df. (mDa)	Df. (ppm)	Iso	DBE
1	82.76	C22 H22 N6 O3 S2	[M+H]+	483.1250	483.1268	-1.8	-3.73	88.82	15.0

Spectra 9. HRMS spectra of compound **6c**

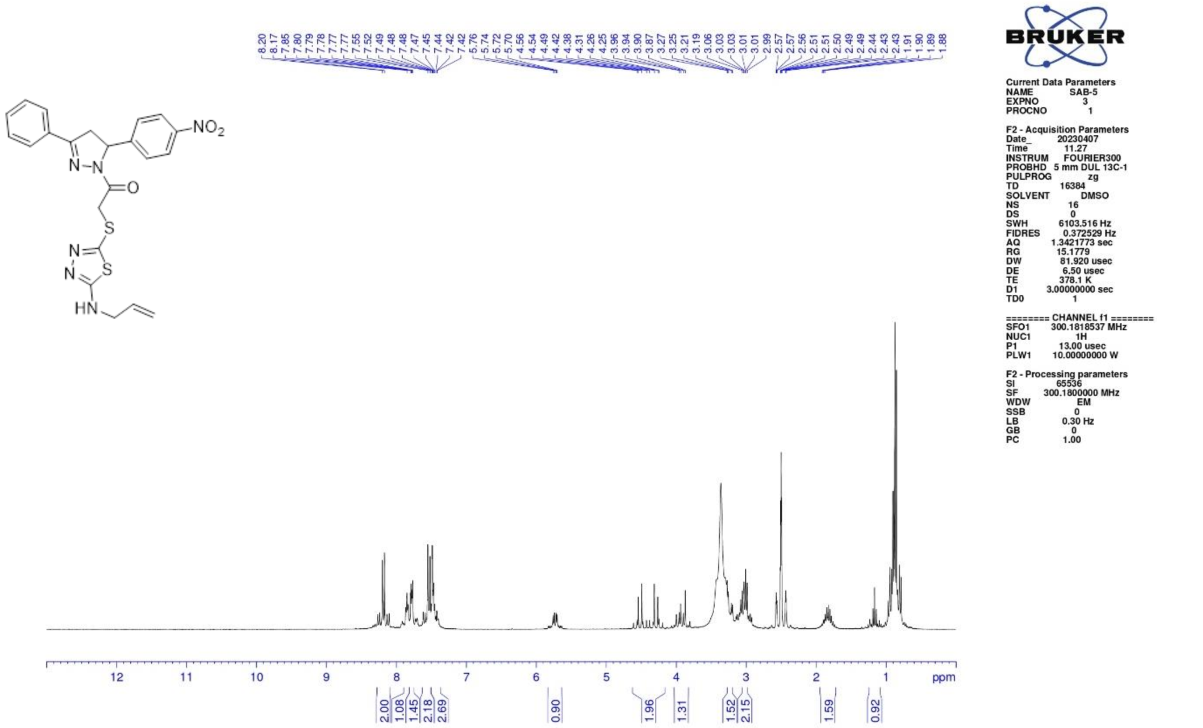

Spectra 10. ^{1}H-NMR spectra of compound **6d**

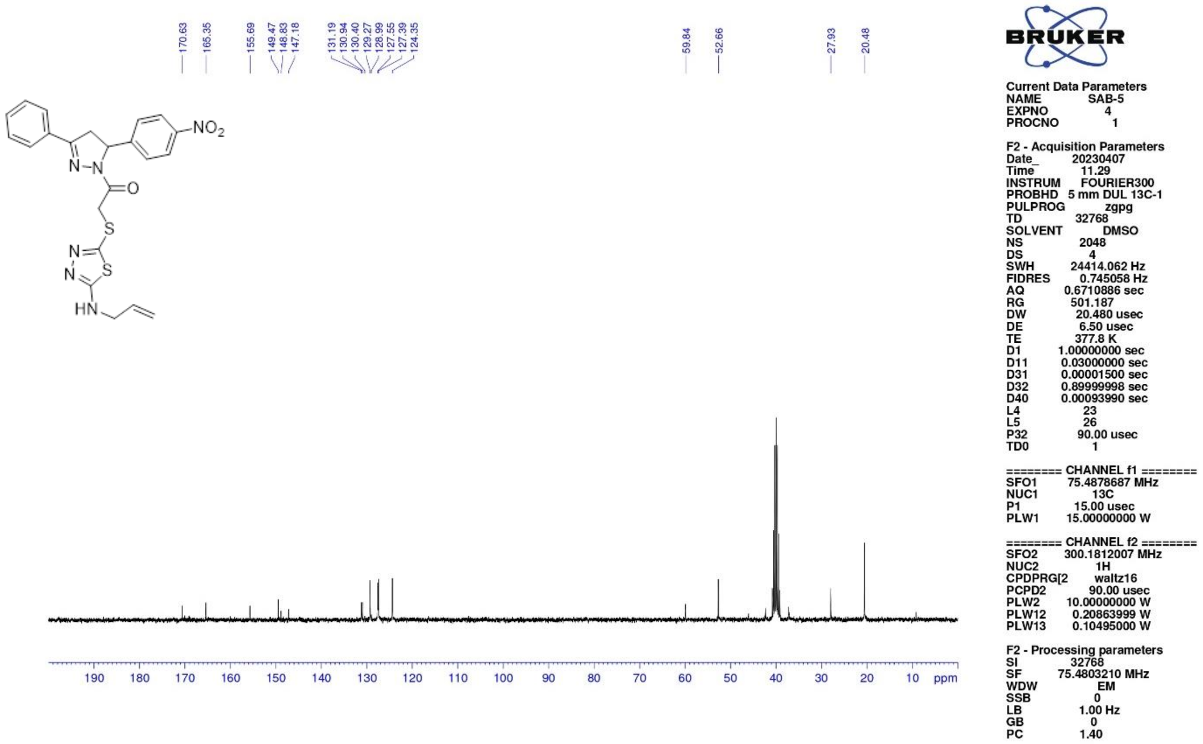

Spectra 11. ^{13}C-NMR spectra of compound **6d**

Data File: C:\LabSolutions\Data\Analiz\derya\SAB-5-5_188.lcd

Elmt	Val.	Min	Max	Elmt	Val.	Min	Max	Elmt	Val.	Min	Max	Elmt	Val.	Min	Max	Use Adduct
H	1	8	40	O	2	0	4	S	2	2	2	Ru	2	0	0	H
C	4	9	40	F	1	0	0	Cl	1	0	0	Pd	2	0	0	
N	3	2	6	P	3	0	0	Br	1	0	0	I	3	0	0	

Error Margin (ppm):	5	DBE Range:	5.0 - 25.0	Electron Ions:	both
HC Ratio:	unlimited	Apply N Rule:	yes	Use MSn Info:	yes
Max Isotopes:	3	Isotope RI (%):	1.00	Isotope Res:	9000
MSn Iso RI (%):	10.00	MSn Logic Mode:	AND	Max Results:	200

Event#: 1 MS(E+) Ret. Time : 3.293 Scan# : 495

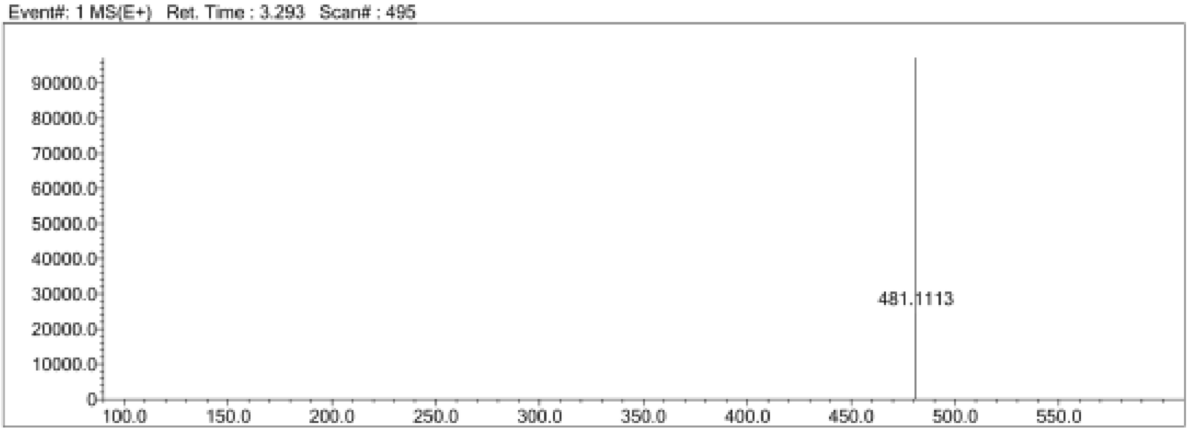

Measured region for 481.1113 m/z

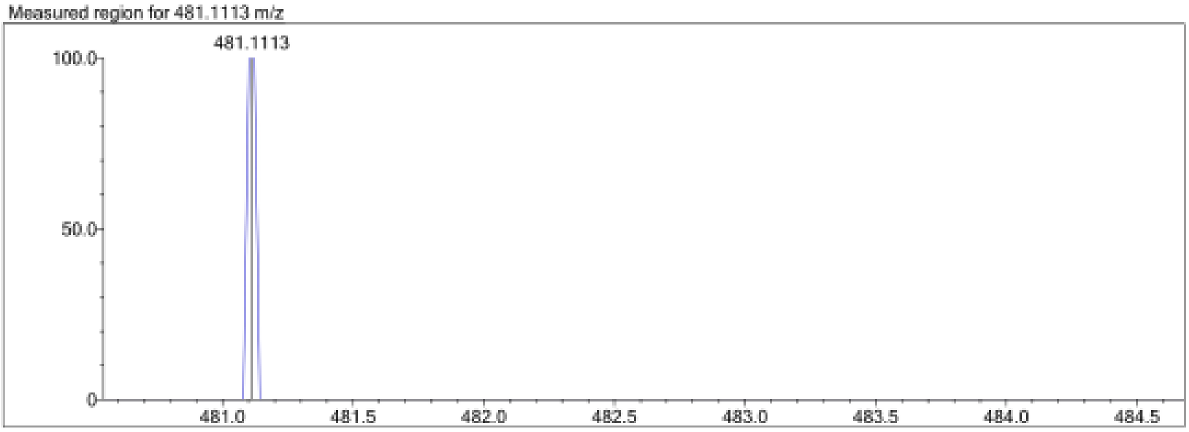

C22 H20 N6 O3 S2 [M+H]+ : Predicted region for 481.1111 m/z

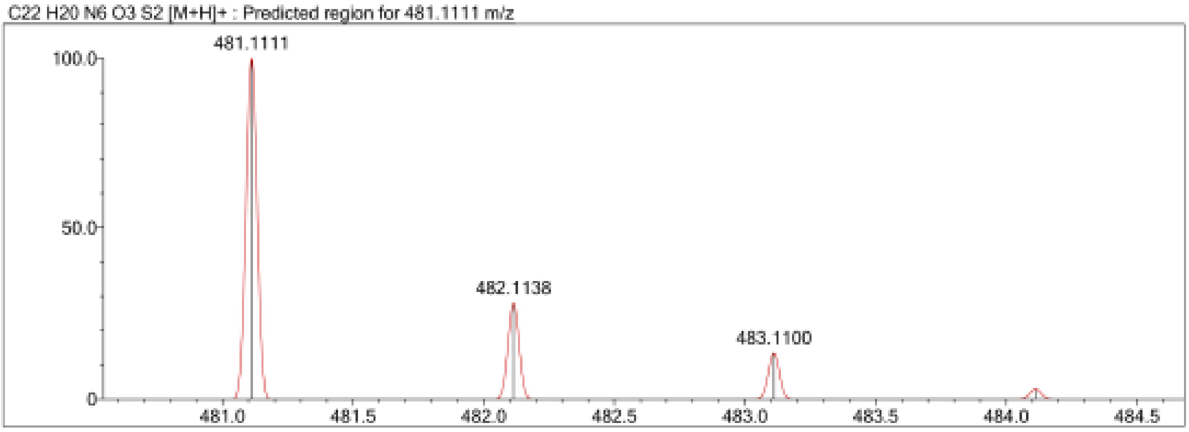

Rank	Score	Formula (M)	Ion	Meas. m/z	Pred. m/z	Df. (mDa)	Df. (ppm)	Iso	DBE
1	0.00	C22 H20 N6 O3 S2	[M+H]+	481.1113	481.1111	0.2	0.42	0.00	16.0

Spectra 12. HRMS spectra of compound **6d**

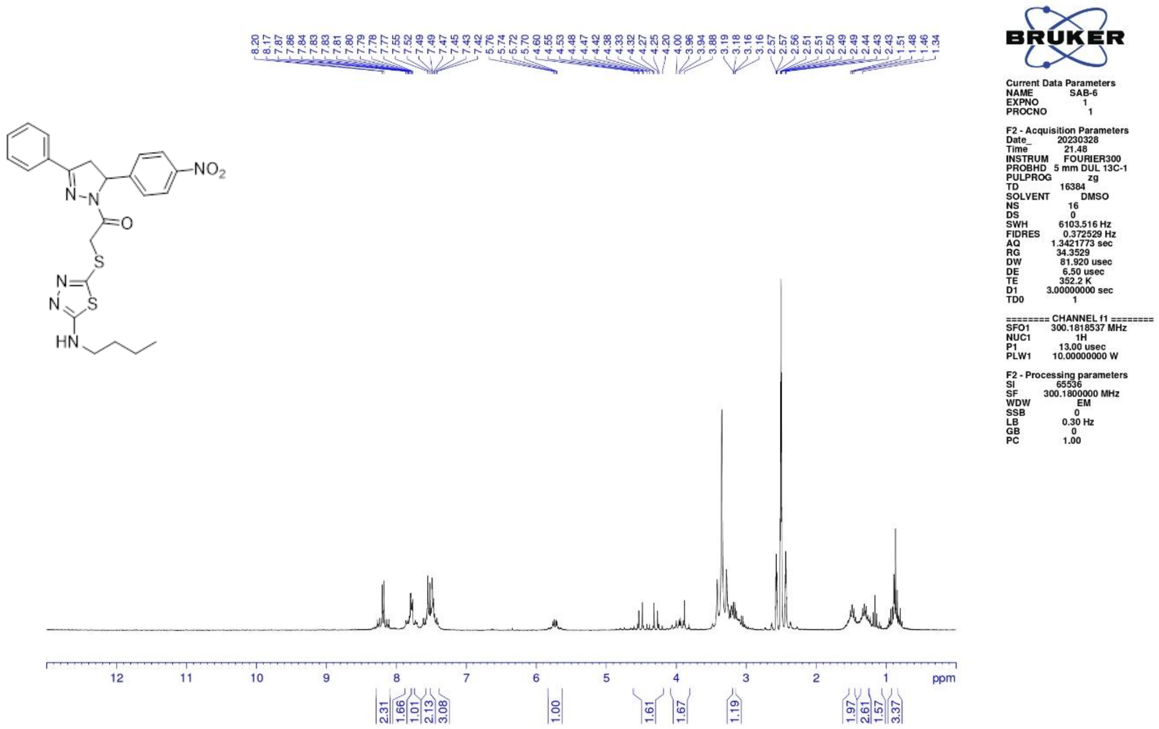

Spectra 13. ^{1}H-NMR spectra of compound **6e**

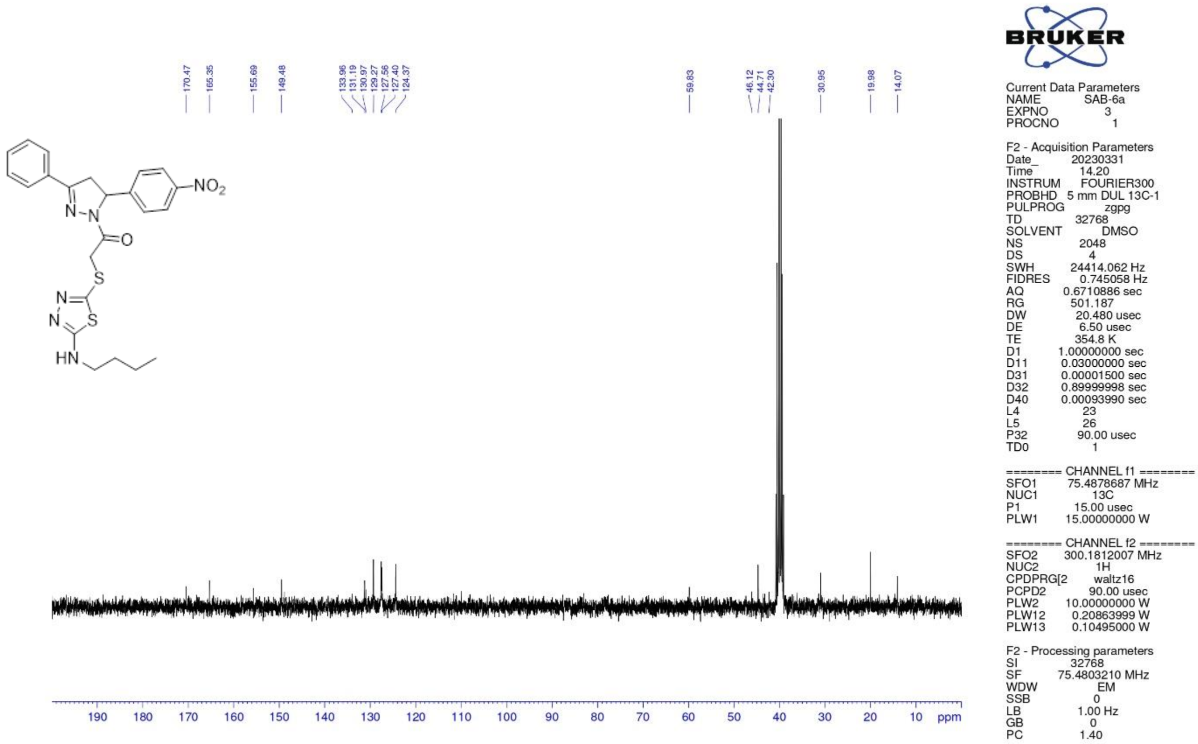

Spectra 14. ^{13}C-NMR spectra of compound **6e**

Formula Predictor Report - SAB-6_189.lcd Page 1 of 1

Data File: C:\LabSolutions\Data\Analiz\derya\SAB-6_189.lcd

Elmt	Val.	Min	Max	Elmt	Val.	Min	Max	Elmt	Val.	Min	Max	Elmt	Val.	Min	Max	Use Adduct
H	1	8	40	O	2	0	4	S	2	2	2	Ru	2	0	0	H
C	4	9	40	F	1	0	0	Cl	1	0	0	Pd	2	0	0	
N	3	2	6	P	3	0	0	Br	1	0	0	I	3	0	0	

Error Margin (ppm):	5	DBE Range:	5.0 - 25.0	Electron Ions:	both
HC Ratio:	unlimited	Apply N Rule:	yes	Use MSn Info:	yes
Max Isotopes:	3	Isotope RI (%):	1.00	Isotope Res:	9000
MSn Iso RI (%):	10.00	MSn Logic Mode:	AND	Max Results:	200

Event#: 1 MS(E+) Ret. Time : 4.213 Scan# : 633

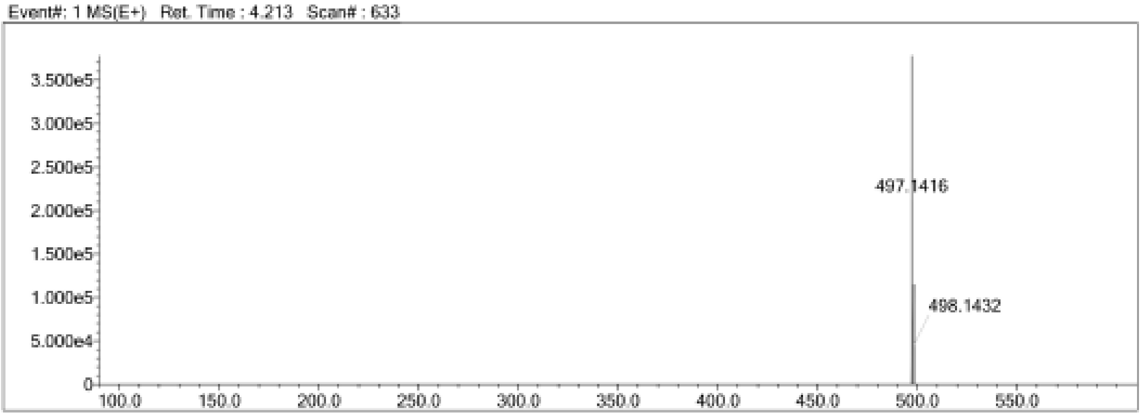

Measured region for 497.1416 m/z

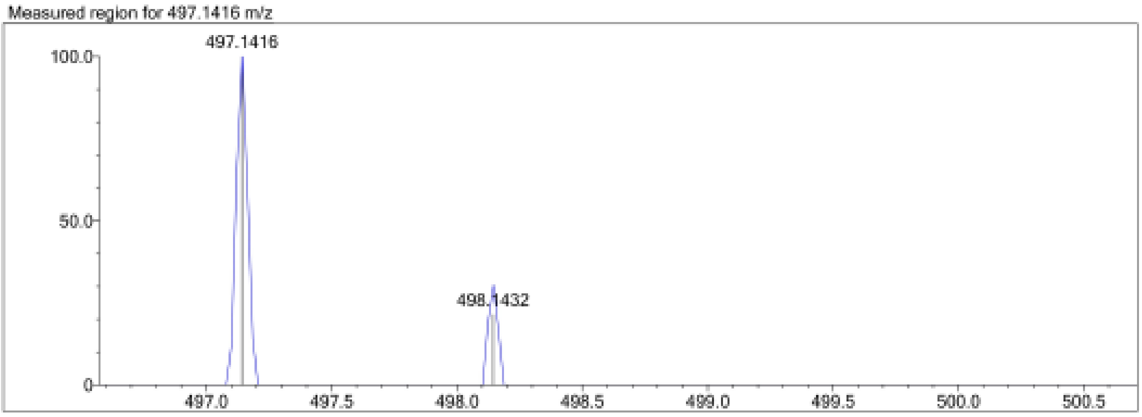

C23 H24 N6 O3 S2 [M+H]+ : Predicted region for 497.1424 m/z

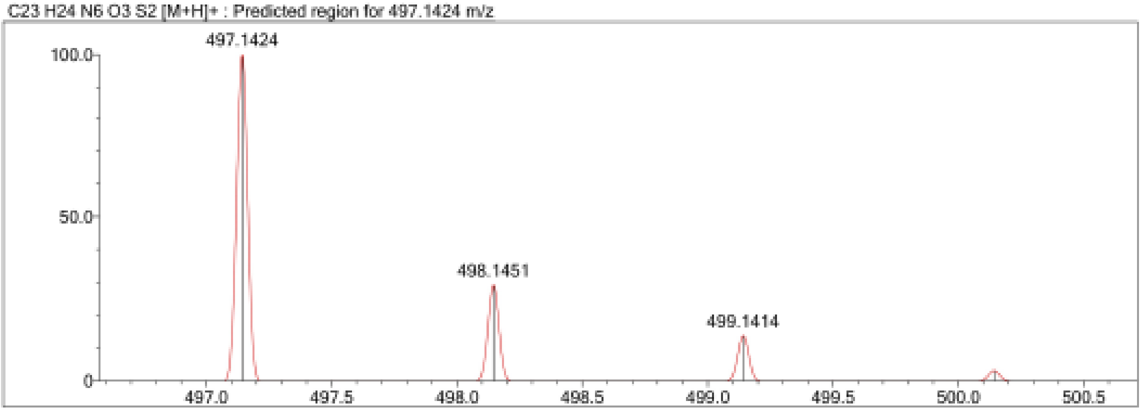

Rank	Score	Formula (M)	Ion	Meas. m/z	Pred. m/z	Df. (mDa)	Df. (ppm)	Iso	DBE
1	0.00	C23 H24 N6 O3 S2	[M+H]+	497.1416	497.1424	-0.8	-1.61	0.00	15.0

Spectra 15. HRMS spectra of compound **6e**

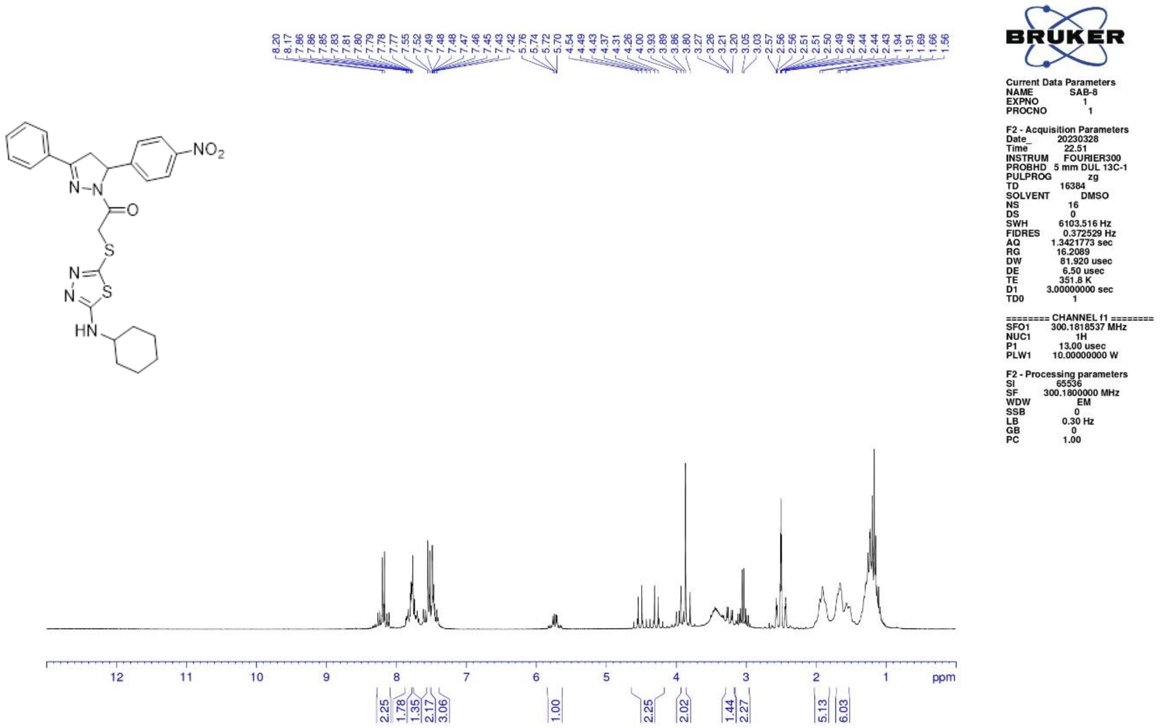

Spectra 16. ^{1}H-NMR spectra of compound **6f**

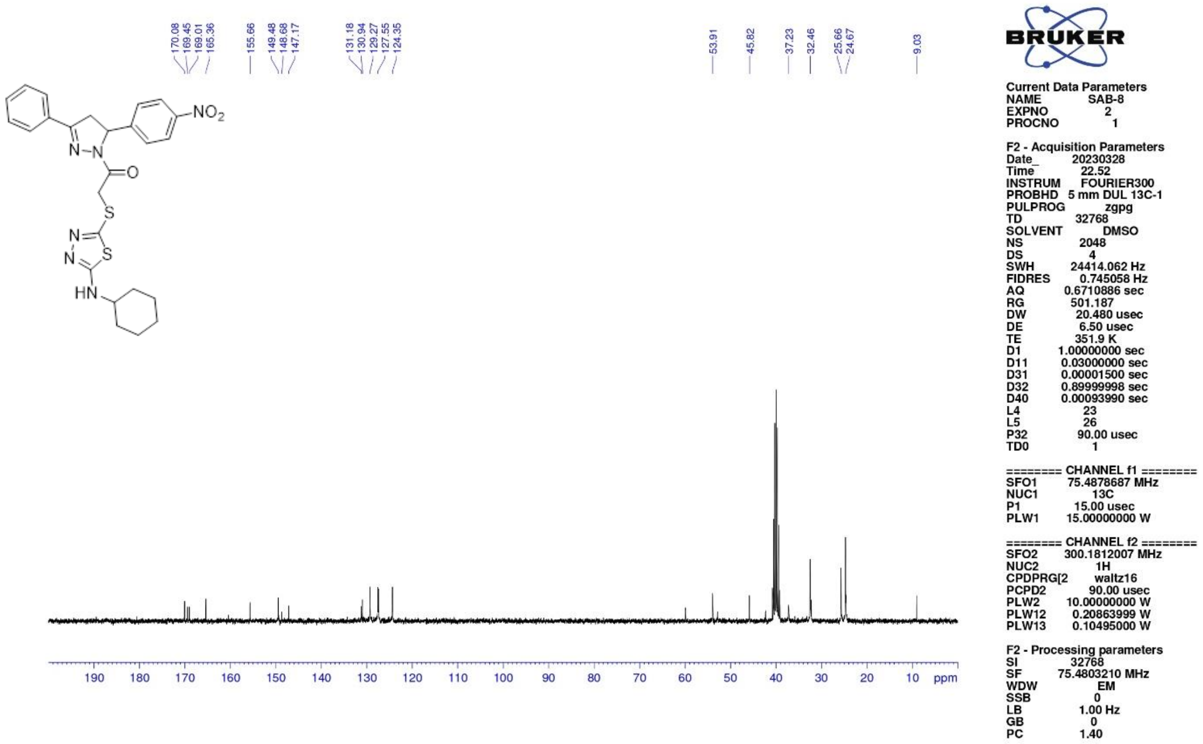

Spectra 17. ^{13}C-NMR spectra of compound **6f**

Data File: C:\LabSolutions\Data\Analiz\derya\SAB-8_190.lcd

Elmt	Val.	Min	Max	Elmt	Val.	Min	Max	Elmt	Val.	Min	Max	Elmt	Val.	Min	Max	Use Adduct
H	1	8	40	O	2	0	4	S	2	2	2	Ru	2	0	0	H
C	4	9	40	F	1	0	0	Cl	1	0	0	Pd	2	0	0	
N	3	2	6	P	3	0	0	Br	1	0	0	I	3	0	0	

Error Margin (ppm):	5	DBE Range:	5.0 - 25.0	Electron Ions:	both
HC Ratio:	unlimited	Apply N Rule:	yes	Use MSn Info:	yes
Max Isotopes:	3	Isotope RI (%):	1.00	Isotope Res:	9000
MSn Iso RI (%):	10.00	MSn Logic Mode:	AND	Max Results:	200

Event#: 1 MS(E+) Ret. Time : 5.240 -> 5.240 Scan# : 787 -> 787

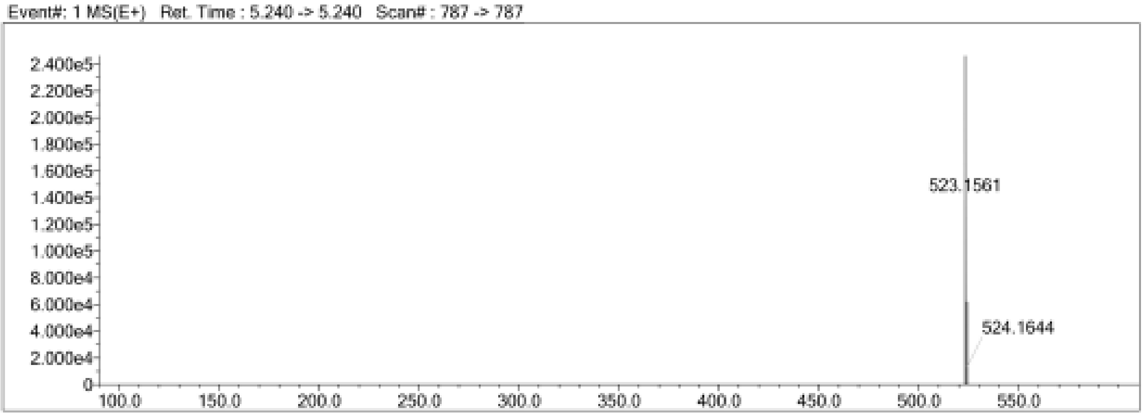

Measured region for 523.1561 m/z

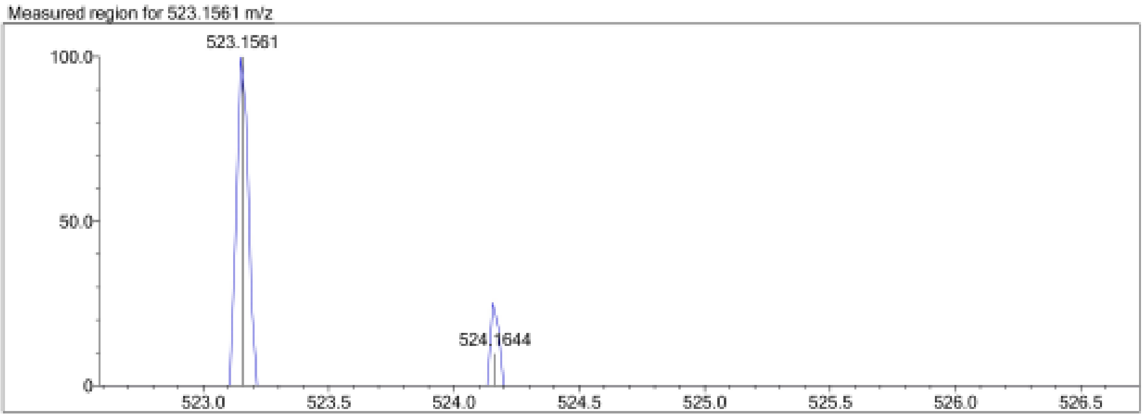

C25 H26 N6 O3 S2 [M+H]+ : Predicted region for 523.1581 m/z

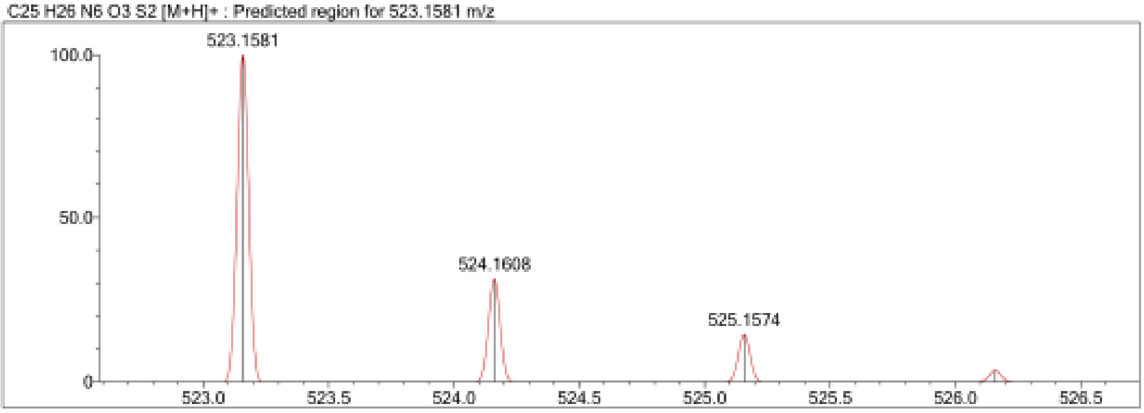

Rank	Score	Formula (M)	Ion	Meas. m/z	Pred. m/z	Df. (mDa)	Df. (ppm)	Iso	DBE
1	0.00	C25 H26 N6 O3 S2	[M+H]+	523.1561	523.1581	-2.0	-3.82	0.00	16.0

Spectra 18. HRMS spectra of compound **6f**

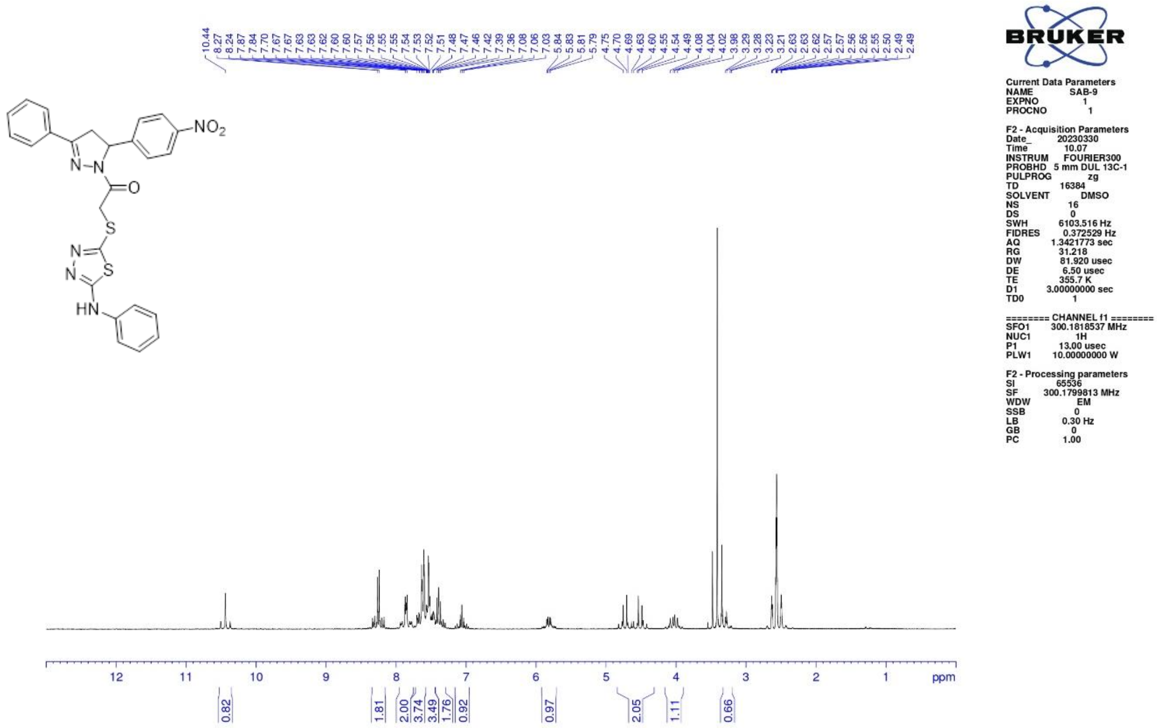

Spectra 19. ^{1}H-NMR spectra of compound **6g**

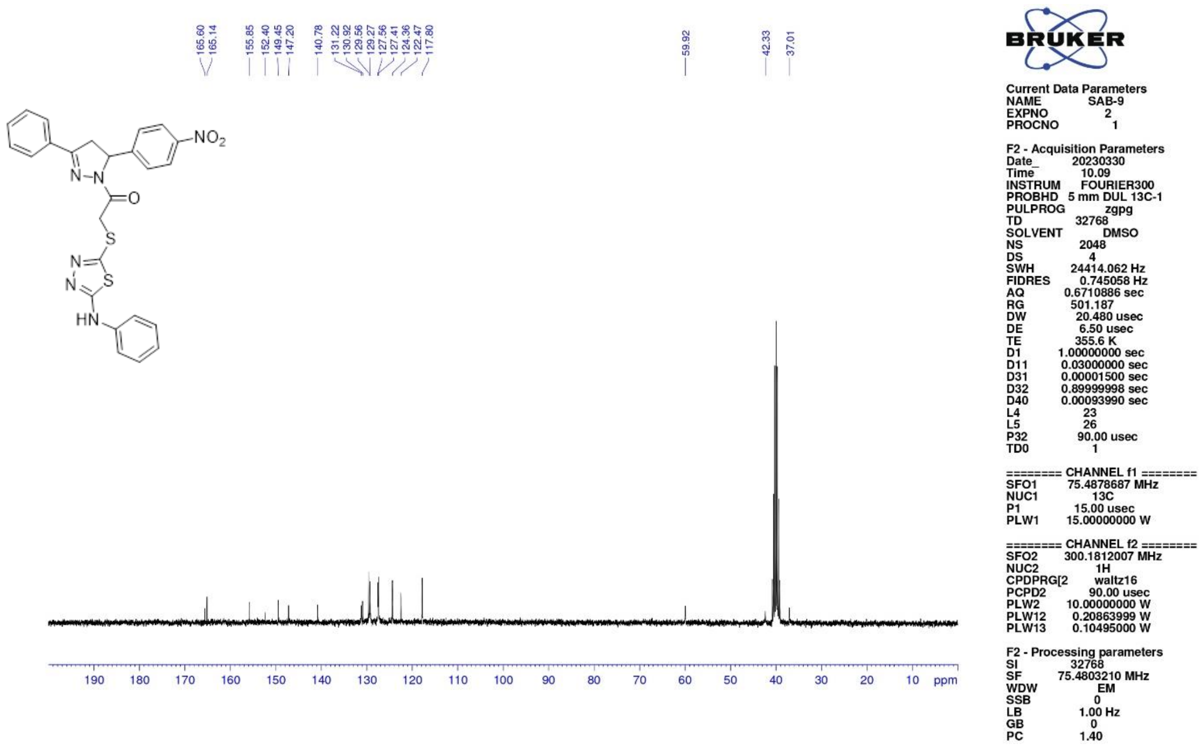

Spectra 20. ^{13}C-NMR spectra of compound **6g**

Data File: C:\LabSolutions\Data\Analiz\derya\SAB-9_191.lcd

Elmt	Val.	Min	Max	Elmt	Val.	Min	Max	Elmt	Val.	Min	Max	Elmt	Val.	Min	Max	Use Adduct
H	1	8	40	O	2	0	4	S	2	2	2	Ru	2	0	0	H
C	4	9	40	F	1	0	0	Cl	1	0	0	Pd	2	0	0	
N	3	2	6	P	3	0	0	Br	1	0	0	I	3	0	0	

Error Margin (ppm):	5	DBE Range:	5.0 - 25.0	Electron Ions:	both
HC Ratio:	unlimited	Apply N Rule:	yes	Use MSn Info:	yes
Max Isotopes:	3	Isotope RI (%):	1.00	Isotope Res:	9000
MSn Iso RI (%):	10.00	MSn Logic Mode:	AND	Max Results:	200

Event#: 1 MS(E+) Ret. Time : 4.627 -> 4.627 Scan# : 695 -> 695

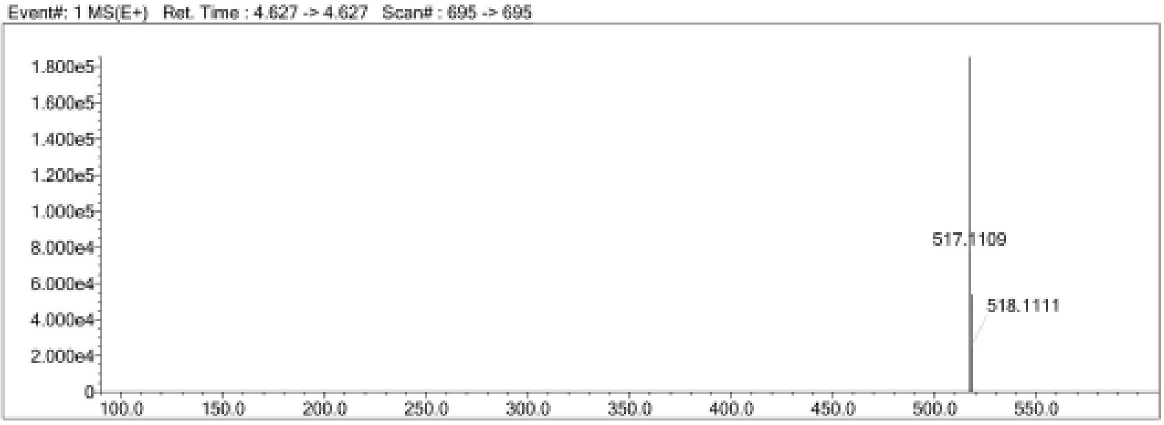

Measured region for 517.1109 m/z

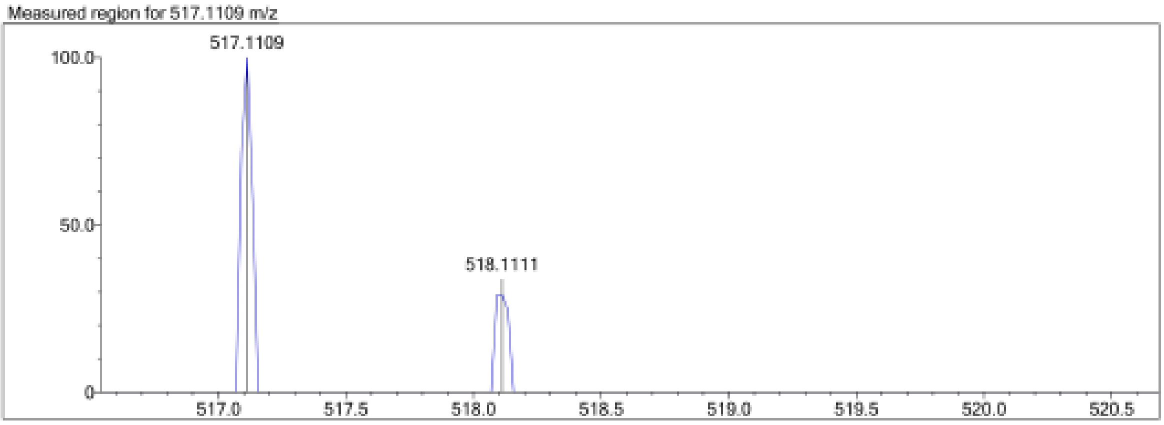

C25 H20 N6 O3 S2 [M+H]+ : Predicted region for 517.1111 m/z

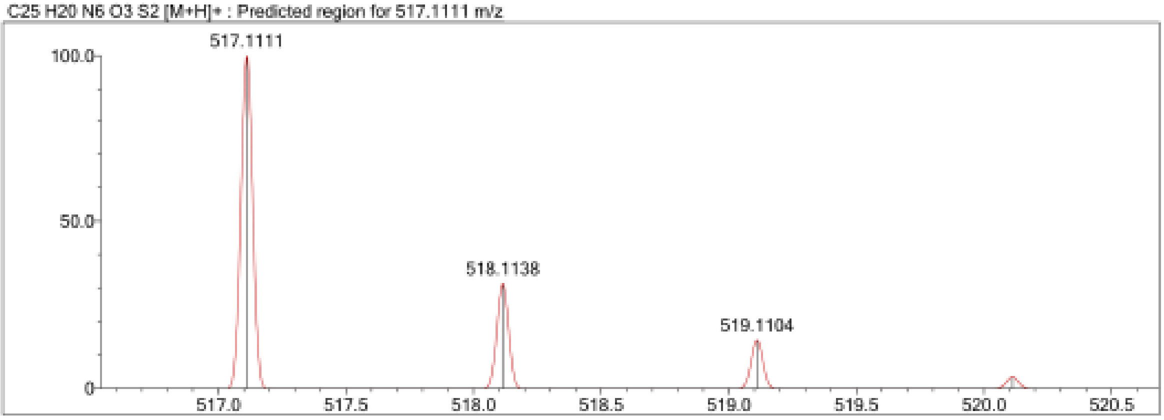

Rank	Score	Formula (M)	Ion	Meas. m/z	Pred. m/z	Df. (mDa)	Df. (ppm)	Iso	DBE
1	0.00	C25 H20 N6 O3 S2	[M+H]+	517.1109	517.1111	-0.2	-0.39	0.00	19.0

Spectra 21. HRMS spectra of compound **6g**

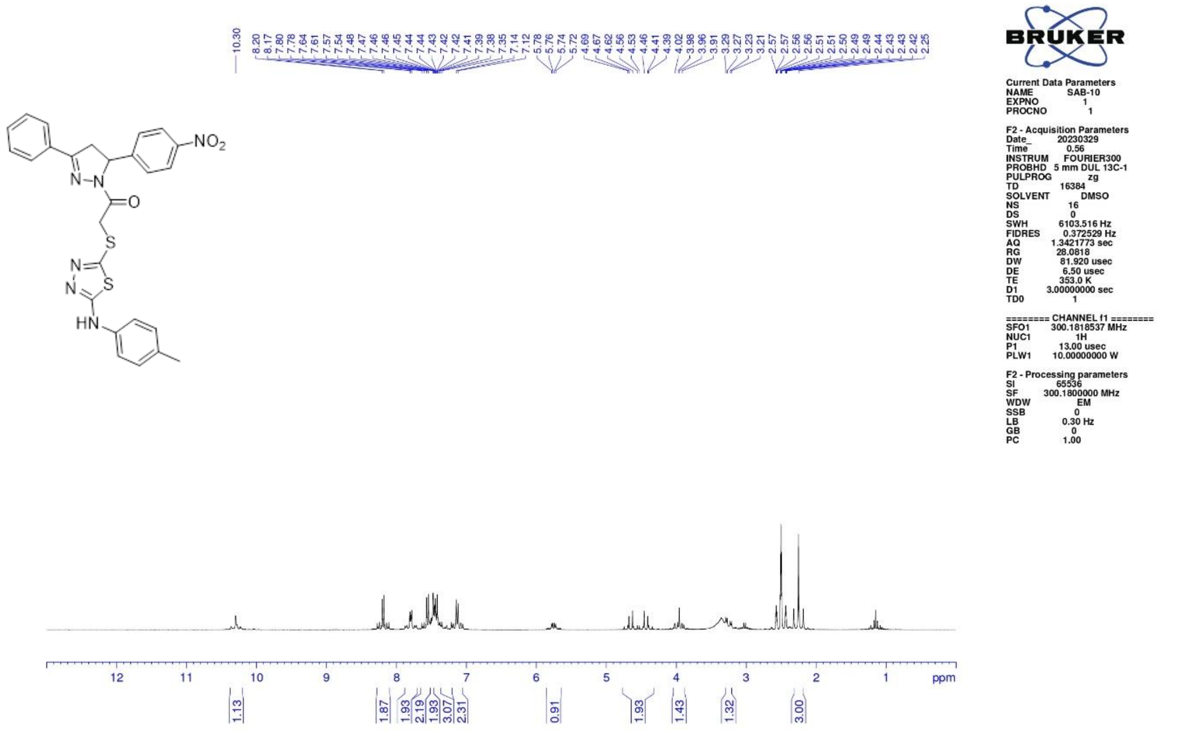

Spectra 22. ^{1}H-NMR spectra of compound **6h**

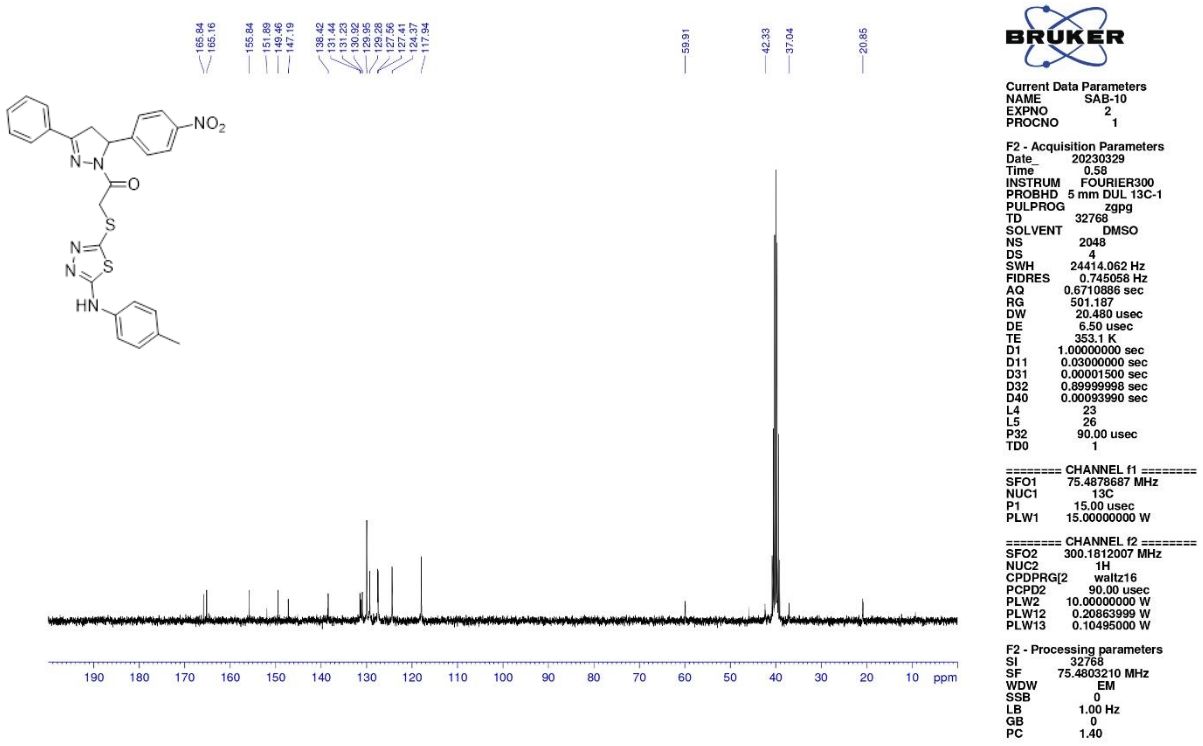

Spectra 23. ^{13}C-NMR spectra of compound **6h**

Data File: C:\LabSolutions\Data\Analiz\derya\SAB-10_192.lcd

Elmt	Val.	Min	Max	Elmt	Val.	Min	Max	Elmt	Val.	Min	Max	Elmt	Val.	Min	Max	Use Adduct
H	1	8	40	O	2	0	4	S	2	2	2	Ru	2	0	0	H
C	4	9	40	F	1	0	0	Cl	1	0	0	Pd	2	0	0	
N	3	2	6	P	3	0	0	Br	1	0	0	I	3	0	0	

Error Margin (ppm):	5	DBE Range:	5.0 - 25.0	Electron Ions:	both
HC Ratio:	unlimited	Apply N Rule:	yes	Use MSn Info:	yes
Max Isotopes:	3	Isotope RI (%):	1.00	Isotope Res:	9000
MSn Iso RI (%):	10.00	MSn Logic Mode:	AND	Max Results:	200

Event#: 1 MS(E+) Ret. Time : 5.547 Scan# : 833

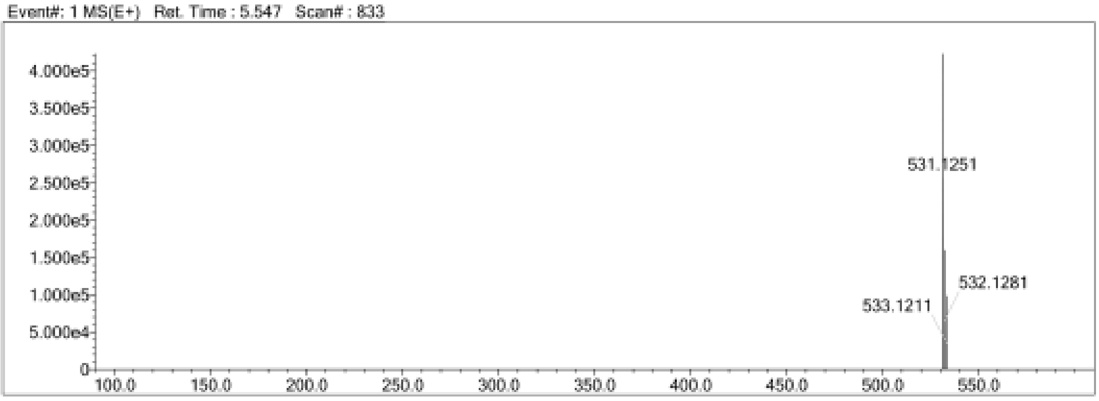

Measured region for 531.1251 m/z

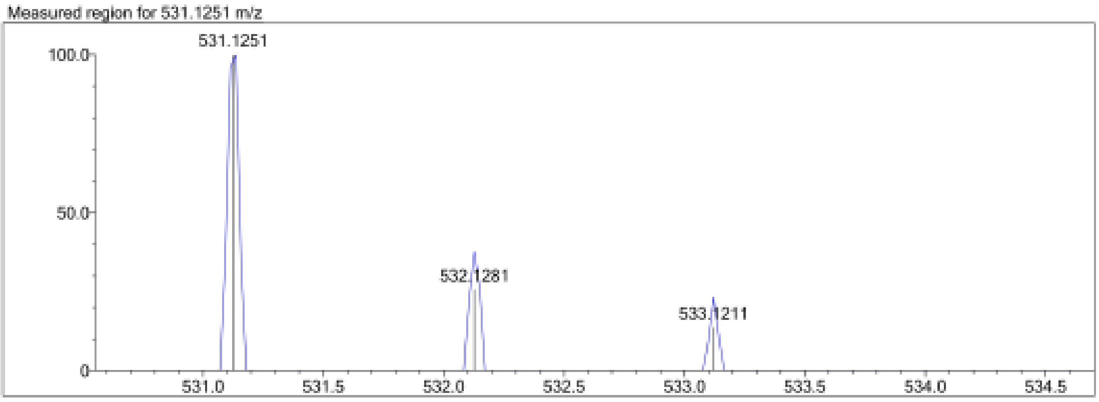

C26 H22 N6 O3 S2 [M+H]+ : Predicted region for 531.1268 m/z

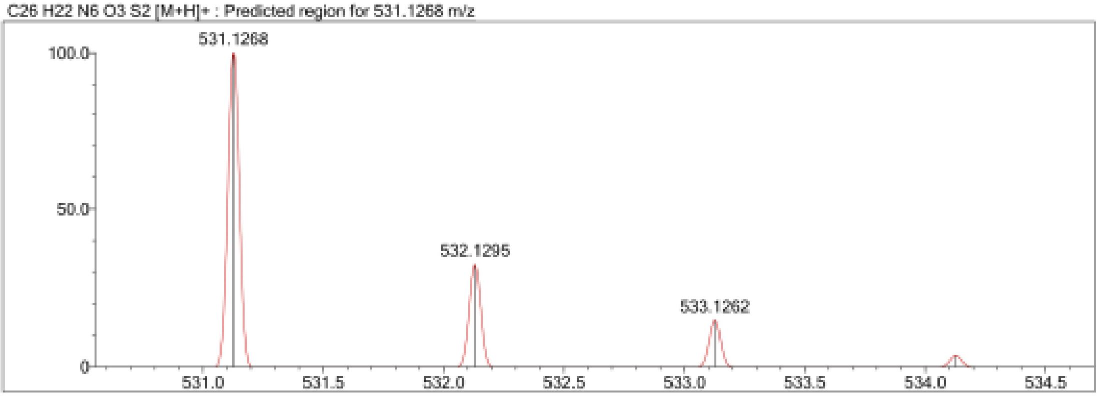

Rank	Score	Formula (M)	Ion	Meas. m/z	Pred. m/z	Df. (mDa)	Df. (ppm)	Iso	DBE
1	71.26	C26 H22 N6 O3 S2	[M+H]+	531.1251	531.1268	-1.7	-3.20	75.41	19.0

Spectra 24. HRMS spectra of compound **6h**

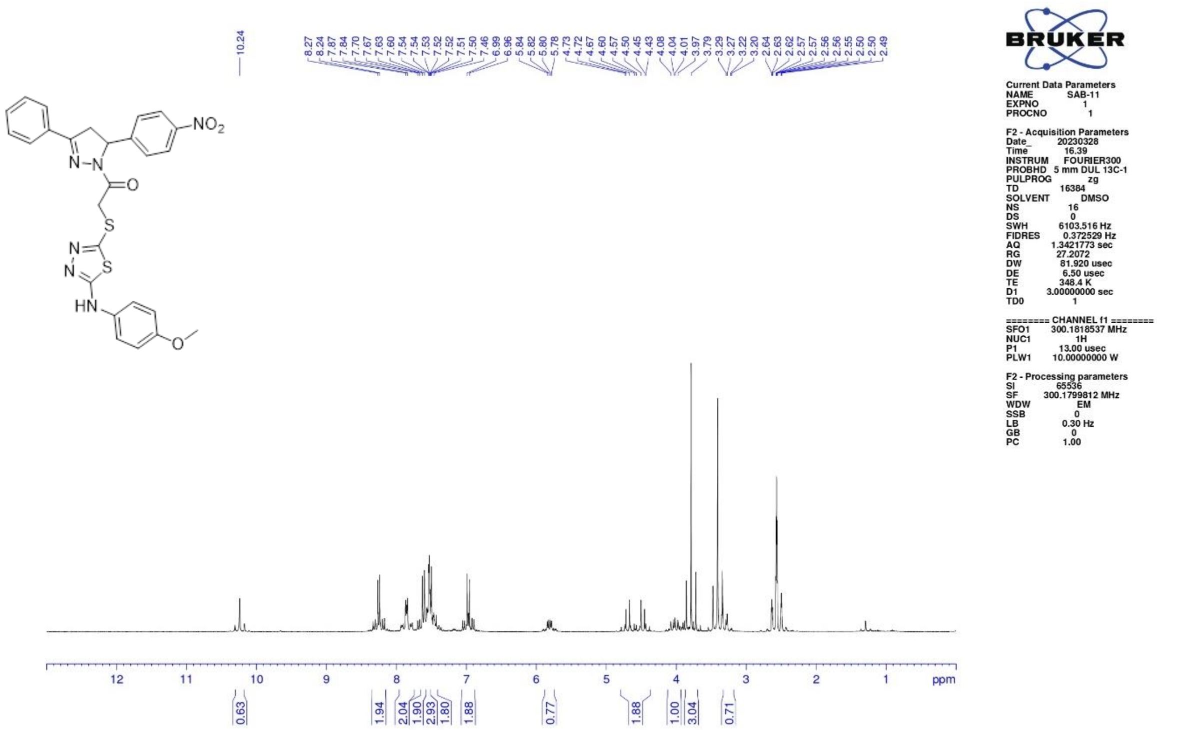

Spectra 25. ^{1}H-NMR spectra of compound **6i**

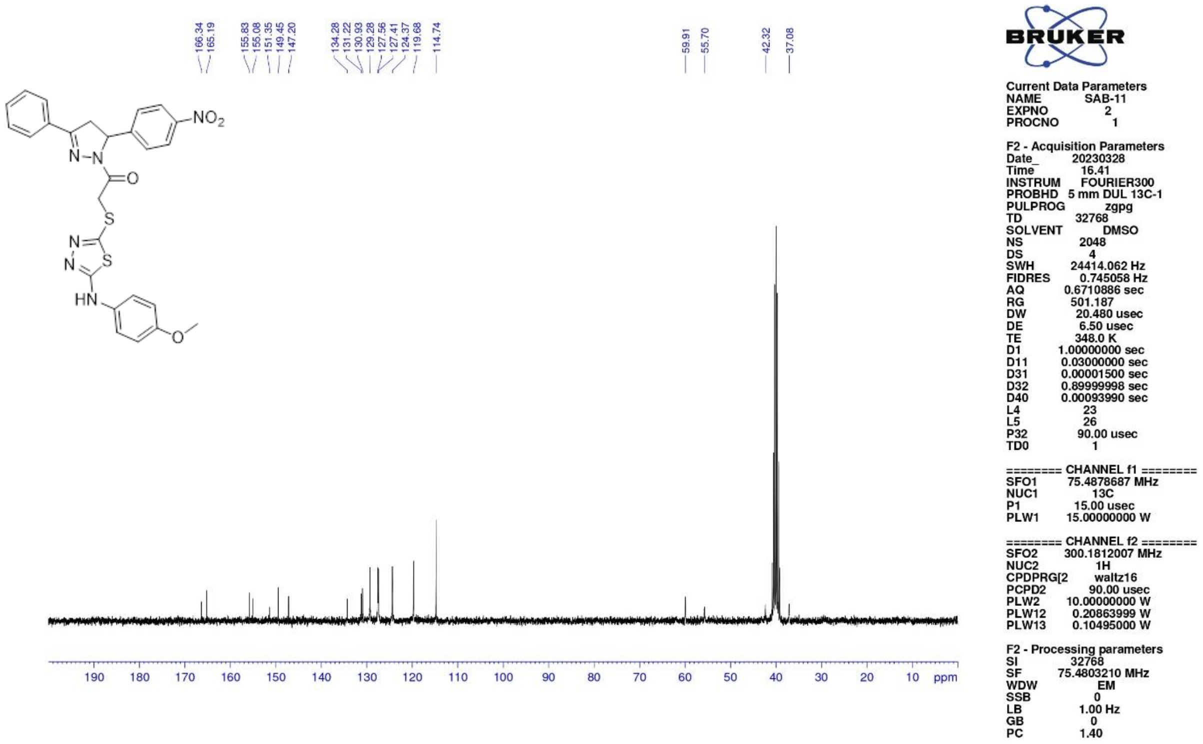

Spectra 26. ^{13}C-NMR spectra of compound **6i**

Data File: C:\LabSolutions\Data\Analiz\derya\SAB-11_193.lcd

Elmt	Val.	Min	Max	Elmt	Val.	Min	Max	Elmt	Val.	Min	Max	Elmt	Val.	Min	Max	Use Adduct
H	1	8	40	O	2	0	4	S	2	2	2	Ru	2	0	0	H
C	4	9	40	F	1	0	0	Cl	1	0	0	Pd	2	0	0	
N	3	2	6	P	3	0	0	Br	1	0	0	I	3	0	0	

Error Margin (ppm):	5	DBE Range:	5.0 - 25.0	Electron Ions:	both
HC Ratio:	unlimited	Apply N Rule:	yes	Use MSn Info:	yes
Max Isotopes:	3	Isotope RI (%):	1.00	Isotope Res:	9000
MSn Iso RI (%):	10.00	MSn Logic Mode:	AND	Max Results:	200

Event#: 1 MS(E+) Ret. Time : 4.213 Scan# : 633

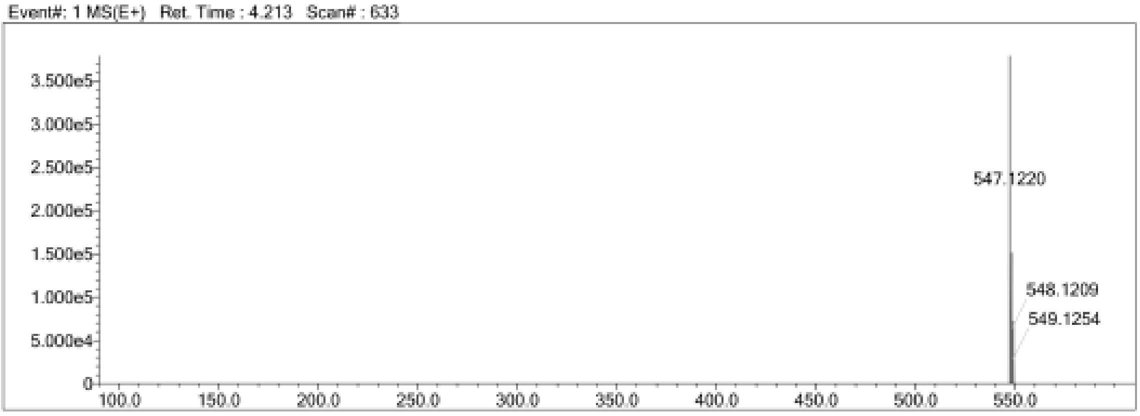

Measured region for 547.1220 m/z

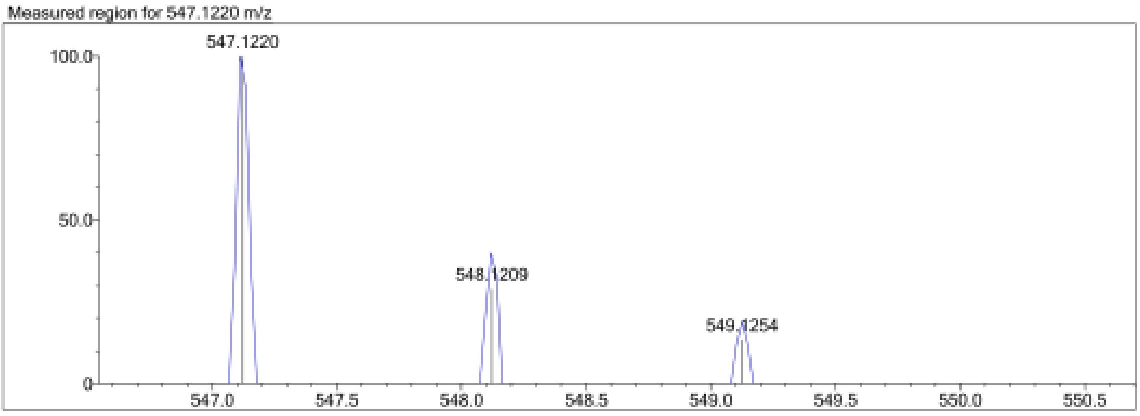

C26 H22 N6 O4 S2 [M+H]+ : Predicted region for 547.1217 m/z

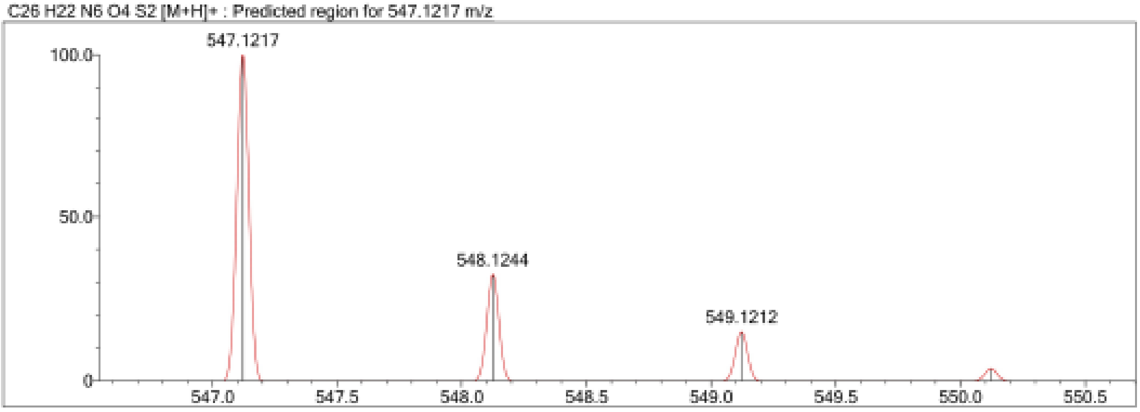

Rank	Score	Formula (M)	Ion	Meas. m/z	Pred. m/z	Df. (mDa)	Df. (ppm)	Iso	DBE
1	75.14	C26 H22 N6 O4 S2	[M+H]+	547.1220	547.1217	0.3	0.55	75.14	19.0

Spectra 27. HRMS spectra of compound **6i**

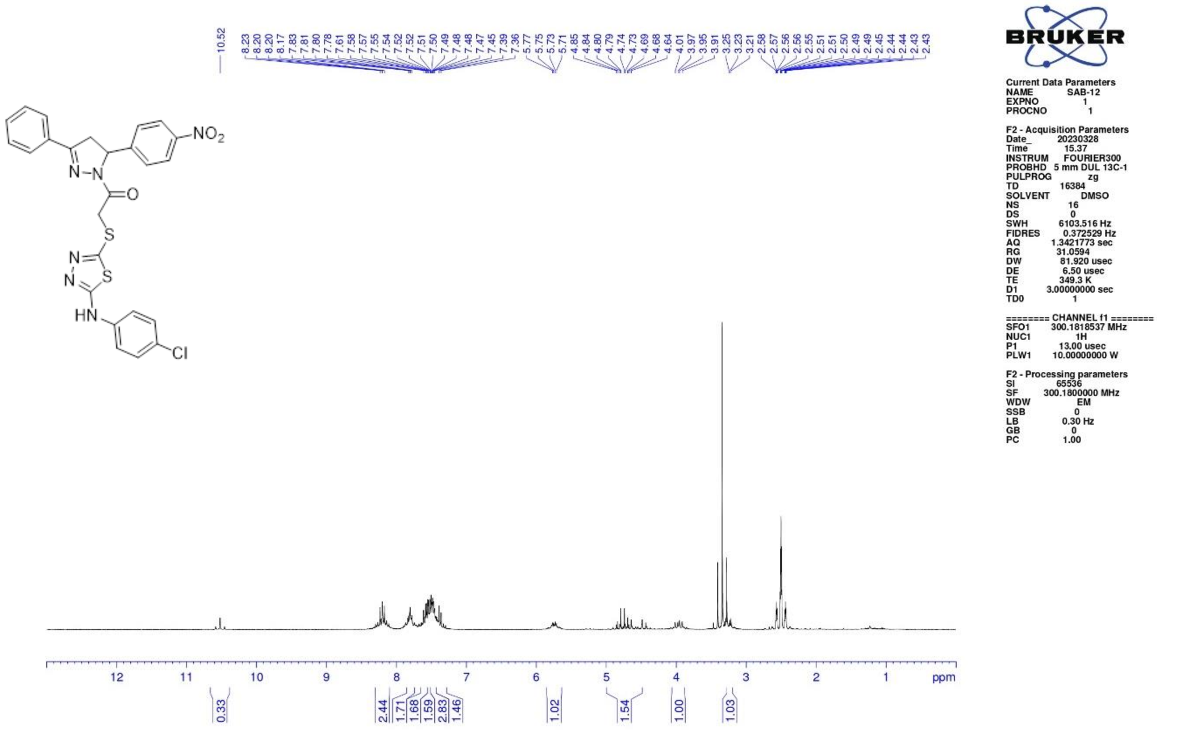

Spectra 28. ^{1}H-NMR spectra of compound **6j**

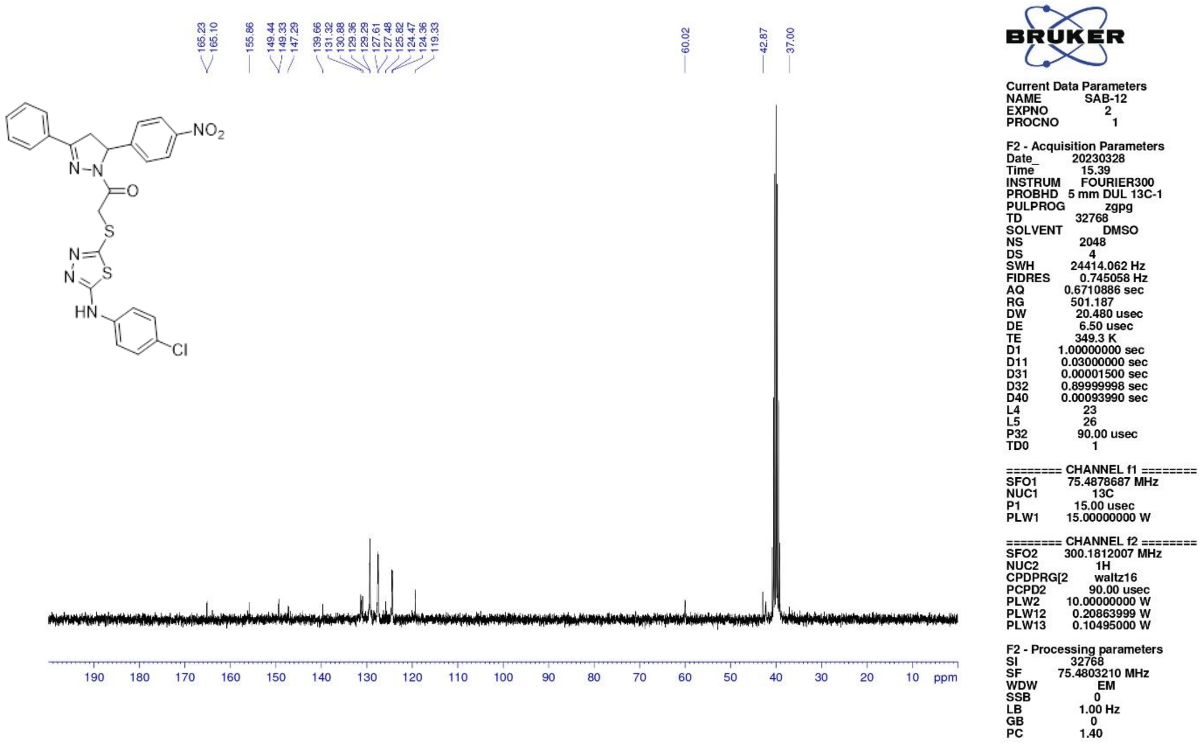

Spectra 29. ^{13}C-NMR spectra of compound **6j**

Data File: C:\LabSolutions\Data\Analiz\derya\SAB-12_194.lcd

Elmt	Val.	Min	Max	Elmt	Val.	Min	Max	Elmt	Val.	Min	Max	Elmt	Val.	Min	Max	Use Adduct
H	1	8	40	O	2	0	4	S	2	2	2	Ru	2	0	0	H
C	4	9	40	F	1	0	0	Cl	1	1	1	Pd	2	0	0	
N	3	2	6	P	3	0	0	Br	1	0	0	I	3	0	0	

Error Margin (ppm):	5	DBE Range:	5.0 - 25.0	Electron Ions:	both
HC Ratio:	unlimited	Apply N Rule:	yes	Use MSn Info:	yes
Max Isotopes:	3	Isotope RI (%):	1.00	Isotope Res:	9000
MSn Iso RI (%):	10.00	MSn Logic Mode:	AND	Max Results:	200

Event#: 1 MS(E+) Ret. Time : 5.907 -> 6.440 Scan# : 887 -> 967

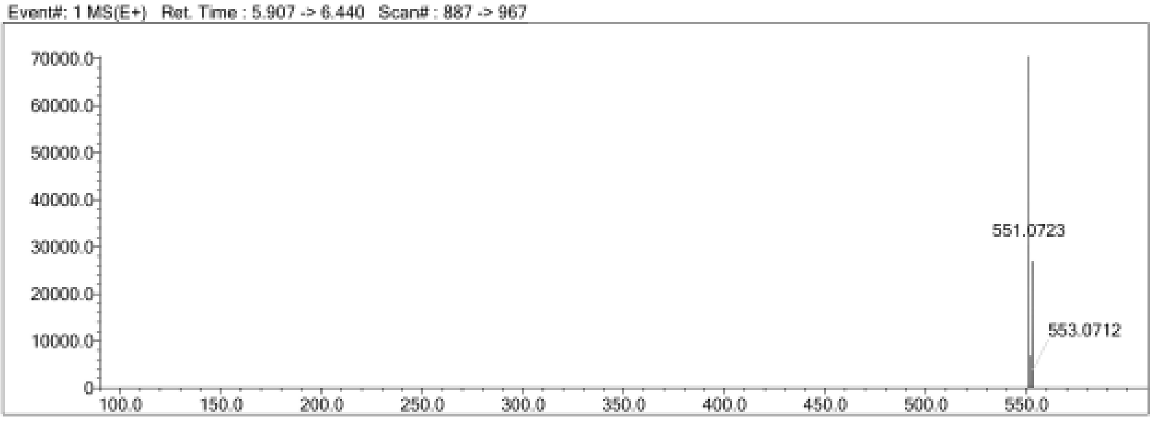

Measured region for 551.0723 m/z

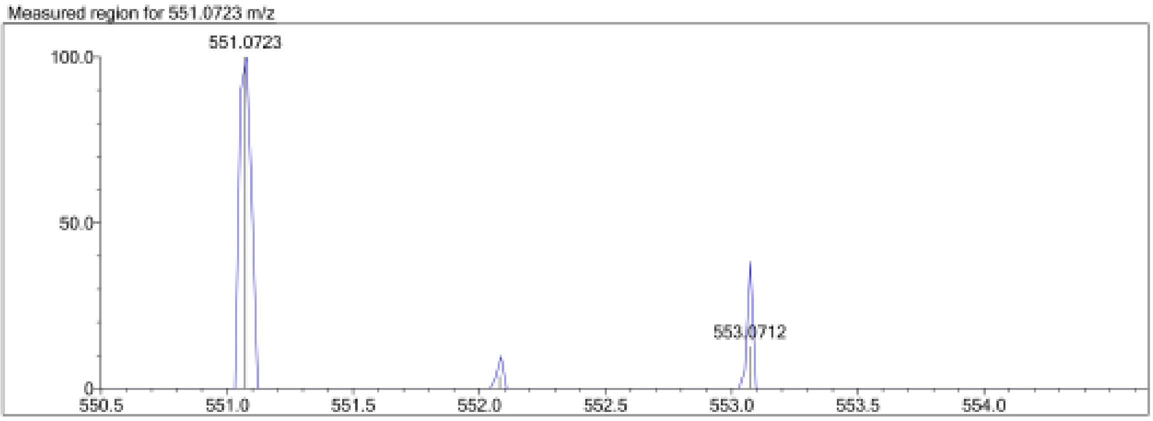

C25 H19 N6 O3 S2 Cl [M+H]+ : Predicted region for 551.0721 m/z

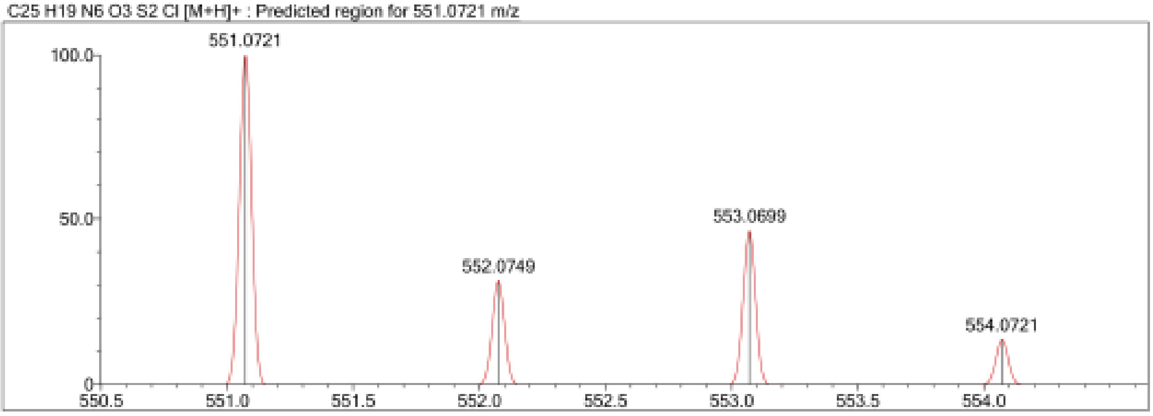

Rank	Score	Formula (M)	Ion	Meas. m/z	Pred. m/z	Df. (mDa)	Df. (ppm)	Iso	DBE
1	37.76	C25 H19 N6 O3 S2 Cl	[M+H]+	551.0723	551.0721	0.2	0.36	37.76	19.0

Spectra 30. HRMS spectra of compound **6j**
